# Irisin protects against vascular calcification by activating autophagy and inhibiting NLRP3-mediated vascular smooth muscle cell pyroptosis in chronic kidney disease

**DOI:** 10.1038/s41419-022-04735-7

**Published:** 2022-03-30

**Authors:** Qi Pang, Peiwen Wang, Yajing Pan, Xingtong Dong, Ting Zhou, Xinyu Song, Aihua Zhang

**Affiliations:** 1 Department of Nephrology, Xuanwu Hospital, Capital Medical University, Changchun Street 45#, 100053 Beijing, China; 2 National Clinical Research Center for Geriatric Disorders, Xuanwu Hospital of Capital Medical University, Beijing, China

**Keywords:** Calcification, Chronic kidney disease

## Abstract

Irisin protects the cardiovascular system against vascular diseases. However, its role in chronic kidney disease (CKD) -associated vascular calcification (VC) and the underlying mechanisms remain unclear. In the present study, we investigated the potential link among Irisin, pyroptosis, and VC under CKD conditions. During mouse vascular smooth muscle cell (VSMC) calcification induced by β-glycerophosphate (β-GP), the pyroptosis level was increased, as evidenced by the upregulated expression of pyroptosis-related proteins (cleaved CASP1, GSDMD-N, and IL1B) and pyroptotic cell death (increased numbers of PI-positive cells and LDH release). Reducing the pyroptosis levels by a CASP1 inhibitor remarkably decreased calcium deposition in β-GP-treated VSMCs. Further experiments revealed that the pyroptosis pathway was activated by excessive reactive oxygen species (ROS) production and subsequent NLR family pyrin domain containing 3 (NLRP3) inflammasome activation in calcified VSMCs. Importantly, Irisin effectively inhibited β-GP-induced calcium deposition in VSMCs in vitro and in mice aortic rings ex vivo. Overexpression of *Nlrp3* attenuated the suppressive effect of Irisin on VSMC calcification. In addition, Irisin could induce autophagy and restore autophagic flux in calcified VSMCs. Adding the autophagy inhibitor 3-methyladenine or chloroquine attenuated the inhibitory effect of Irisin on β-GP-induced ROS production, NLRP3 inflammasome activation, pyroptosis, and calcification in VSMCs. Finally, our in vivo study showed that Irisin treatment promoted autophagy, downregulated ROS level and thereby suppressed pyroptosis and medial calcification in aortic tissues of adenine-induced CKD mice. Together, our findings for the first time demonstrated that Irisin protected against VC via inducing autophagy and inhibiting VSMC pyroptosis in CKD, and Irisin might serve as an effective therapeutic agent for CKD-associated VC.

## Introduction

Cardiovascular events are the leading cause of death in patients with chronic kidney disease (CKD). Vascular calcification (VC) in the medial layer of the vessel wall is a prominent feature in CKD patients [1, 2]. Clinical studies have shown that the prevalence of VC increases with decreasing kidney function in CKD patients, and patients with end-stage renal disease have an even higher risk of cardiovascular events and mortality [3, 4]. Since there is no effective treatment for VC of CKD patients at present, developing strategies to suppress VC process is essential.

Pyroptosis is a pro-inflammatory form of programmed cell death accompanied by rapid plasma membrane rupture and intracellular contents release [5]. Accumulating evidence suggests that pyroptosis may contribute to several diseases, including infectious diseases [6], cancer [7], neurodegenerative disorders [8], and cardiovascular disease [9]. Different from apoptosis, pyroptosis is mainly mediated by inflammasome-dependent caspase1 (CASP1) activation. Activated CASP1 is responsible for processing and maturing interleukin 1 beta/18 (IL1B/18) and cleaving gasdermin D (GSDMD) to release the N‐terminal fragments of GSDMD (GSDMD-N); these fragments then form membrane pores to induce the release of inflammatory factors [5–9]. Previous research has revealed upregulation of the NLR family pyrin domain containing 3 (NLRP3) inflammasome in artery specimens from CKD patients [10] and high phosphate-treated vascular smooth muscle cells (VSMCs), and inhibition of NLRP3 inflammasome activation prevented VSMC calcification, suggesting a critical role of the NLRP3 inflammasome in the pathogenesis of VC [10, 11]. Most importantly, the executor of pyroptosis (activated CASP1), which is induced by the NLRP3 inflammasome, is increased in calcified VSMCs [10, 11]. However, whether pyroptosis is involved in VSMC calcification and how it is related to the formation of VC remain to be clarified.

Recently, autophagic removal of NLRP3 inflammasome activators [12, 13], NLRP3 inflammasome components [14], and cytokines [15], has been shown to reduce pyroptosis and inflammatory cytokine release in diverse disease states. Irisin, a recently identified myokine, is secreted from skeletal muscle during exercising and plays an important role in maintaining the biological function of various cells and tissues [16]. Some of its beneficial properties have been attributed to autophagy induction, suggesting that irisin is an important regulator of autophagy [17]. Our previous study showed that lower serum Irisin levels were associated with an increased risk of VC in peritoneal dialysis patients [18] and hemodialysis patients [19], indicating a potential relationship between the circulating Irisin concentration and VC in CKD patients. However, the role of Irisin in CKD‐associated VC has not yet been studied.

In the present study, we aimed to investigate the role of VSMC pyroptosis in CKD-related VC and to further determine whether Irisin could exert protective effects against VC by inducing autophagy and inhibiting the pyroptosis pathway. In addition, we evaluated the therapeutic potential of Irisin in adenine‐induced CKD mice. Taken together, these findings may provide new targets and strategies to prevent the progression of VC in CKD.

## Materials and methods

### VSMC Culture and Treatment

The mouse vascular smooth muscle cell line (MOVAS-1) was obtained from the American Type Culture Collection (ATCC, Manassas, VA, USA). Cells were cultured in DMEM (Hyclone, Logan, UT, USA) containing 10% FBS (Gibco, Waltham, MA, USA) and 1% (v/v) penicillin/streptomycin at 37 °C in a humidified incubator with 5% CO_2_. Cells between passages 6 and 8 were used for all experiments. VSMC calcification was induced according to previous protocols [20, 21]. Briefly, VSMCs were cultured with growing medium in the presence of β-glycerophosphate (β-GP) (10 mM; G9422; Sigma, St. Louis, MO, USA) for 7 days. To investigate the role of Irisin in VSMC calcification, Irisin (067-29; Phoenix Pharmaceuticals Inc, Burlingame, CA, USA) at different concentrations (50 or 100 ng/ml dissolved in PBS) was used to treat VSMCs in the presence of β-GP medium for 7 days with medium changes every 2–3 days. For pharmacological treatment, the CASP1 inhibitor VX-765 (10 μM; HY-13205), NLRP3 inhibitor MCC950 (100 μM; HY-12815A), ROS scavenger N-acetyl-L-cysteine (NAC, 20 μM; HY-B0215), autophagy inducer rapamycin (200 nM; HY-10219), autophagy inhibitor 3-methyladenine (3-MA, 5 mM; HY-19312), and chloroquine (CQ, 25 μM; HY-17589A) were purchased from MedChem Express (NJ, USA) and used to treat VSMCs according to the experimental design.

### Determination of Calcification

For Alizarin red staining, the calcified VSMCs were washed with PBS 3 times and then fixed in 4% paraformaldehyde (PFA) for 20 min at room temperature (RT). After washing with PBS, VSMCs were exposed to 2% Alizarin red solution (Sigma, PH = 4.2) for 5 min at 37 °C. Excess Alizarin red reagent was removed by washing with distilled water and observing under a light microscope (Nikon, Tokyo, Japan). For artery calcification staining, paraffin sections from the mouse thoracic aorta were dehydrated and then stained with Alizarin red solution for 5 min. The calcium nodules were observed under a light microscope.

For Von Kossa staining [21, 22], paraffin sections from the mouse thoracic aorta were dehydrated and then immersed in 1% silver nitrate solution for 1 h under strong sunlight. Next, the sections were placed in 5% sodium thiosulfate solution for 5 min to remove unreacted silver and counterstained with aldehyde-fuchsin. The calcium nodules were then observed under a light microscope.

For measuring the calcium content, mice aortic tissues and VSMC lysates were prepared as previously described [22]. The calcium content was measured using the QuantiChrom Calcium Assay Kit (Biosino Bio‐Technology and Science, Beijing, China) according to the manufacturer’s instructions. The results were normalized to the total protein concentration.

### Western blotting

Protein lysates were obtained from mice aortic tissues and cultured VSMCs using RIPA buffer (P0013, Beyotime Biotechnology, Shanghai, China) supplemented with complete protease and phosphatase inhibitor cocktail, and then the protein concentration was measured using a BCA protein assay kit (P0010, Beyotime). Equal amounts of protein were separated on 10% or 15% SDS–polyacrylamide gels and transferred to polyvinylidene fluoride membranes (Millipore, Billerica, MA, USA). After blocking with 5% nonfat milk for 1 h at RT, the membranes were incubated with the primary antibodies at 4 °C overnight. After washing with PBST 3 times, the membranes were incubated with horseradish peroxidase (HRP)‐labeled rabbit (1:2000; ab205718) or mouse (1:2000; ab6789) secondary antibodies for 2 h at RT. Finally, the membrane was washed with PBST 3 times, and protein bands were detected using an ECL reagent (Pierce, Rockford, IL, USA). If required, membranes were stripped using a commercial western blot stripping buffer (Applygen Technologies, Beijing, China), and then were incubated with other antibodies. For quantitative analysis [23], band intensity was quantified using ImageJ software. The relative density was the ratio of the intensity of the target gene to that of GAPDH. Primary antibodies against NLRP3 (1:1000; ab270449), and CASP1 (1:1000; ab179515) were purchased from Abcam (Cambridge, MA, USA). Antibodies against PYCARD/ASC (1:1000; 67824), GSDMD (1:1000; 10137), IL1B (1:1000;63124), MAP1LC3B/LC3B (1:1000; 2775), SQSTM1 (1:1000; 23214) and GAPDH (1:1000; 97166) were purchased from Cell Signaling Technology (CST, Danvers, MA, USA).

### Quantitative real-time polymerase chain reaction (qRT-PCR)

Total RNA was extracted from cultured VSMCs using TRIzol reagent (Invitrogen, Carlsbad, CA, USA), and cDNA was synthesized using a reverse transcription system kit (RR036A; Takara, Dalian, China) according to the manufacturer’s instructions. Next, qRT-PCR was performed using SYBR Green Premix Ex Taq^TM^ (RR820A; Takara) and an ABI prism 7500 sequence detection system (Applied Biosystems, Foster City, California, USA). *Gapdh* was used as an internal control for normalization. Relative quantification was performed according to the 2^−ΔΔCt^ method, and each sample was analyzed at least 3 times. The PCR primer sequences used in this study are listed in Supplementary Table S1.

### Plasmids and cell transfection

*Nlrp3* overexpression plasmids (pcDNA 3.1- *Nlrp3*) were designed and constructed by Genechem Company (Shanghai, China). Empty pcDNA 3.1 plasmids were used as the negative control. When VSMCs reached approximately 70–80% confluence, the cells were transfected with different plasmids using Lipofectamine 2000 (Invitrogen) according to the manufacturer’s instructions. Transfection efficacy was assessed by western blotting and qRT-PCR.

### Reactive oxygen species (ROS) assay

The ROS levels in VSMCs and mice aortic tissues were detected using an ROS assay kit (S0033; Beyotime). Briefly, VSMCs and aorta sections were incubated with DCFH-DA (10 μM) at 37 °C for 30 min in the dark. The ROS levels in VSMCs were analyzed using a FACSCalibur flow cytometer (BD Bioscience, USA), and the data analysis was performed using the FlowJo software program. The ROS levels in aortic sections were visualized using confocal microscopy (Olympus, Heidelberg, Germany).

### Immunofluorescence staining

#### VSMCs

VSMCs were seeded on coverslips in 12-well plates and incubated overnight. After the designated treatment, the cells were fixed with 4% PFA for 20 min and permeabilized with 0.2% Triton X-100 for 30 min at RT. Next, the cells were blocked with 5% bovine serum albumin for 1 h and incubated with the primary antibody against CASP1 (1:100; 22915-1-AP; Proteintech, Wuhan, China) at 4 °C overnight, followed by incubation with secondary antibody (1:1000; donkey anti-rabbit Alexa Fluor 594, ab150076; Abcam) in the dark for 1 h at RT. The nuclei were stained with DAPI (ab104139; Abcam) for 5 min and visualized using confocal microscopy. For quantitative analysis of immunofluorescence intensity, images were processed using ImageJ software as described previously [24, 25]. First, region of interest was selected for the measurement of area and mean fluorescent intensity (MFI). Second, the background intensity was measured by selecting three distinct areas in the background with no staining. Finally, average background fluorescence was subtracted from raw MFI values.

#### Analysis of frozen aorta tissue slides

Frozen aorta tissue sections were fixed with 4% PFA for 15 min and permeabilized with 0.2% Triton X-100 for 30 min at RT. Next, the sections were blocked with 5% bovine serum albumin for 1 h and incubated with primary antibodies against NLRP3 (1:100; bs-10021R; Bioss, Beijing, China), CASP1(1:100; Proteintech) or SQSTM1 (1:100; CST) at 4 °C overnight. The sections were co-stained with ACTA2/α-SMA (1:100; A5228; Sigma) to identify VSMC segments in mice aortic tissues. After washing with PBS, the sections were incubated with the appropriate secondary antibodies in the dark for 1 h at RT. The nuclei were stained with DAPI for 5 min and visualized using confocal microscopy.

### Supernatant ELISA detection

The IL1B levels in VSMC culture supernatants and mouse serum were determined using commercial assay kits (ab100704; Abcam) according to the manufacturer’s protocols.

### Analysis of DNA fragmentation (TUNEL assays)

TUNEL staining was performed to detect DNA fragmentation of VSMCs using a detection kit (Roche, USA) according to the manufacturer’s instructions. Briefly, VSMCs were cultured on coverslips in 12-well plates overnight. After the designated treatment, the cells were fixed with 4% PFA for 20 min and permeabilized with 0.2% Triton X-100 for 30 min at RT. Subsequently, the cells were incubated with the TUNEL reaction mixture at 37 °C in the dark for 1 h at RT. The nuclei were stained with DAPI for 5 min, and the slides were viewed using confocal microscopy. The nuclei of TUNEL-positive cells have been stained green, and the nuclei of DAPI cells have been stained blue. For quantification of TUNEL-positive VSMCs [26, 27], at least 10 fields with more than 200 cells were randomly selected from three different experiments per group to estimate the apoptosis percentage. The index of apoptosis was expressed as the ratio of positively stained apoptotic cells to the total number of VSMCs counted × 100%.

### Pyroptotic cell death assay (Hoechst 33342/PI and LDH release)

To confirm pore formation in cell membranes, Hoechst 33342 and propidium iodide (PI) staining were performed. VSMCs were seeded on coverslips in 12-well plates overnight. After the designated treatment, the cells were incubated with a mixed solution of Hoechst 33342 and PI (P0137, Beyotime) for 25 min at RT and photographed under a fluorescence microscope. For LDH release, VSMCs were seeded into 6-well plates overnight. After the indicated treatment, the cell culture supernatants were collected, and LDH release was measured using an LDH Kit (Jiancheng Bioengineering Institute, Nanjing, China) according to the manufacturer’s instructions.

### Autophagy flux analysis

VSMCs were transfected with mRFP-GFP-LC3 double-labeled adenovirus (Hanbio Technology, Shanghai, China) according to the manufacturer’s protocol (MOI = 50). After adenovirus transfection for 48 h, the adenovirus-containing supernatant was removed and the cells were washed with PBS 3 times. After being treated with experimental conditions as indicated, cells were washed with PBS and were fixed with 4% PFA for 20 min at RT. Finally, the fluorescence images were captured by confocal microscopy. Autophagic flux was evaluated by calculating the number of yellow (autophagosomes) and red (autolysosomes) puncta as previously described [28–30].

### Animals and Treatment

#### Animal model

6-week-old male C57BL/6 J mice (18–20 g) were purchased from the National Model Animal Center of Nanjing University and maintained in a specific pathogen-free environment with free access to food and water and a 12-h light/dark cycle. The mice were randomly divided into a control group (*n* = 8), CKD model group (*n* = 8), and CKD model + Irisin group (*n* = 8). Control mice were fed a standard chow diet (containing 1.2% calcium and 0.6% phosphate), while CKD model mice were fed a chow diet containing 0.25% adenine (A8626; Sigma) for 4–6 weeks as previously described [31]. After 6 weeks, the blood urea nitrogen (BUN) and creatinine (Cr) levels were monitored to evaluate a mouse model of CKD. To induce media calcification, the CKD mice were then fed a high calcium and phosphate diet (3% calcium and 1.8% phosphate) and administered calcitriol (active vitamin D3; 1 μg/kg; twice a week; Sigma) subcutaneously for 12–16 weeks [22].

#### Drug treatments

Irisin was dissolved in sterile normal saline (NS). The mice in the CKD model + Irisin group were treated with purified Irisin (2 µg in 100 µl of NS per mouse; twice a week) by tail vein injection for 12–16 weeks, and the mice in the CKD model group received an equivalent volume of NS in the same manner [32]. At the end of the animal experiment, the mice were sacrificed, and blood samples were collected to determine the BUN, Cr, calcium, and phosphate levels using microplate test kits (Nanjing Jiancheng Bioengineering Institute) according to the manufacturer’s instructions. ELISA kits were used to determine the serum concentrations of Irisin (Elabscience, Wuhan, China) according to the manufacturer’s instructions. Abdominal aorta samples were collected for further analysis.

#### Aortic ring calcification

Aortas (from the thoracic to the iliac arteries) were isolated from 8-week-old male mice in a sterile manner as previously described [11, 20]. The vessels were cut into 2- to 3-mm rings and then incubated in medium containing β-GP (10 mM) with or without Irisin for 7 days, with medium changes every 2–3 days.

#### Animal ethics

All animal experiments were performed according to the National Institutes of Health Guide for the Care and Use of Laboratory Animals and approved by the Animal Care and Use Committees of Capital Medical University Animal Experiments Ethics Committee.

### Statistical analysis

All the data were expressed as means ± standard error of the mean (SEM). GraphPad Prism 6.0 software was used for statistical analysis. Comparisons between two groups were performed using Student’s *t-*test. One-way analysis of variance (ANOVA) followed by Tukey’s post hoc test was performed when comparisons among multiple groups. Each experiment was repeated three times. A *P* value <0.05 was considered statistically significant.

## Results

### CASP1-mediated pyroptotic cell death is involved in VSMC calcification

**Fig. 1:**
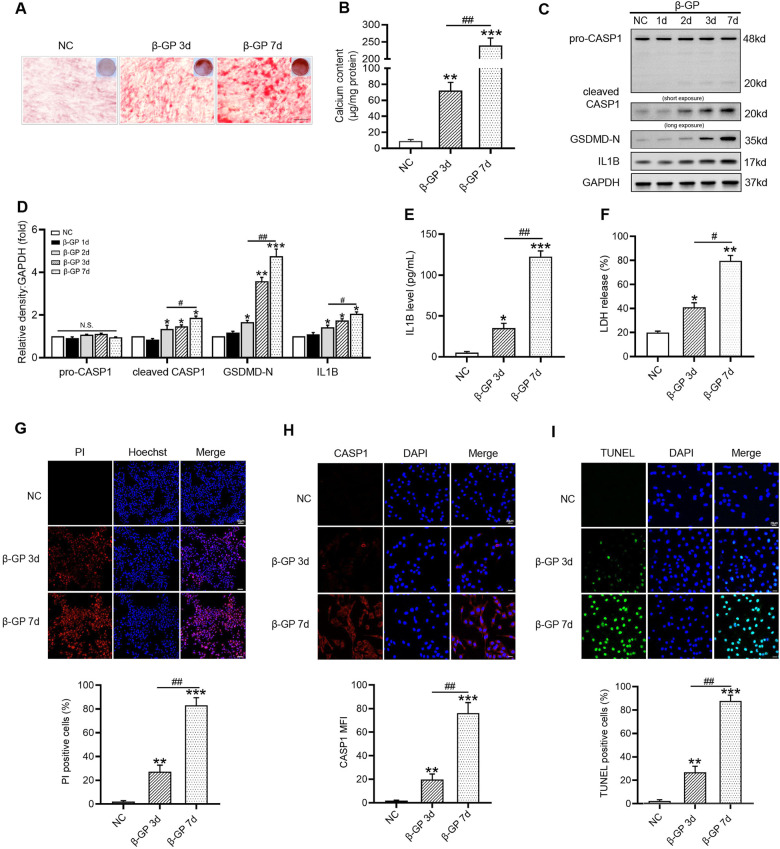
Pyroptosis occurs in VSMCs after exposure to β-GP. VSMCs were treated with β-GP (10 mM) for the indicated time. **A** Calcium deposition in VSMCs was assessed by Alizarin red staining (positive staining: red; scale bar = 100 μm). **B** Quantitative analysis of calcium deposition in VSMCs normalized to the protein content. **C** Protein levels of pro-CASP1, cleaved CASP1, GSDMD-N, and IL1B were determined by western blotting. **D** Quantification of the results shown in C. **E** The IL1B content in VSMC culture supernatants was determined by ELISA. **F** The release of LDH was detected using the LDH Assay Kit. **G** The percentage of PI-positive cells was measured using Hoechst 33342 (blue)/PI (red) double staining (top: Representative images; bottom: Quantitative analysis of PI-positive cells). **H** CASP1 expression was determined by immunofluorescent staining (top: Representative images; bottom: Mean fluorescence intensity (MFI) of CASP1 was quantified). **I** The percentage of TUNEL-positive cells (green) was evaluated by TUNEL staining (top: Representative images; bottom: Quantitative analysis of TUNEL-positive cells). Scale bar = 50 μm for (**G**); Scale bar = 20 μm for (**H**) and (**I**). Data are expressed as mean ± SEM. *P < 0.05, **P < 0.01, ***P < 0.001 vs. control group; ^*#*^*P* < 0.05, ^*##*^*P* < 0.01 vs. indicated group; and N.S. not significant.

VSMCs were cultured in medium containing β-GP for 7 days, and increased extracellular calcium deposition was proven by alizarin red staining (Fig. 1A) and calcium content assays (Fig. 1B). During VSMC calcification, the protein levels of pyroptosis-related markers, including cleaved CASP1, GSDMD-N, and IL1B, were significantly increased as early as Day 2 and further increased at Day 7 compared with those of the controls (Fig. 1C, D). Subsequently, we detected IL1B production in the culture medium. As expected, the IL1B level was obviously upregulated after β-GP treatment (Fig. 1E). Moreover, LDH release (Fig. 1F) and PI-positive cells (Fig. 1G) were markedly elevated in β-GP-treated VSMCs, suggesting that the plasma membrane was ruptured and became leaky. In addition, immunofluorescence staining for CASP1 (Fig. 1H) and TUNEL (Fig. 1I) staining revealed that β-GP significantly increased the levels of cell pyroptosis. These results strongly suggested that pyroptotic cell death occurred in β-GP-treated VSMCs in vitro VC model. Thus, we chose a treatment concentration of 10 mM and duration of 7 days for β-GP in subsequent experiments.

**Fig. 2:**
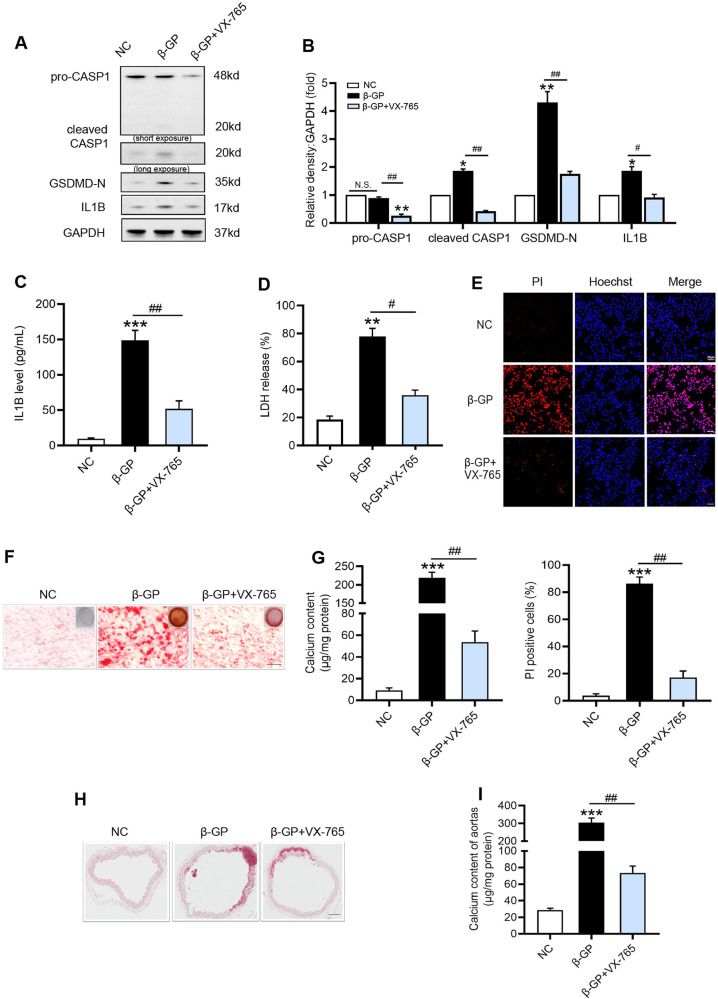
Inhibition of CASP1 attenuates β-GP-induced VSMC pyroptosis and calcification. VSMCs were incubated in culture medium containing β-GP (10 mM) for 7 days with or without CASP1 inhibitor (VX-765; 10 μM). **A** Protein levels of pro-CASP1, cleaved CASP1, GSDMD-N, and IL1B were determined by western blotting. **B** Quantification of the results shown in A. **C** The IL1B content in VSMC culture supernatants was determined by ELISA. **D** The release of LDH was detected using the LDH Assay Kit. **E** The percentage of PI-positive cells was measured using Hoechst 33342 (blue)/PI (red) double staining (top: Representative images; bottom: Quantitative analysis of PI-positive cells). Scale bar = 50 μm. **F** Calcium deposition in VSMCs was assessed by Alizarin red staining (positive staining: red; scale bar = 100 μm). **G** Quantitative analysis of calcium deposition in VSMCs normalized to the protein content. Mouse aortic rings were incubated in the culture medium containing β-GP (10 mM) for 7 days with or without VX-765(10 μM). Calcification was assessed by Alizarin red staining (**H**), and calcium content of aortas was measured as described in methods (**I**). (positive staining: red; scale bar = 100 μm). Data are expressed as mean ± SEM. *P < 0.05, **P < 0.01, ***P < 0.001 vs. control group; ^*#*^*P* < 0.05, ^*##*^*P* < 0.01 vs. indicated group; and N.S. not significant.

Next, our results showed that CASP1 inhibitor (VX-765) lowered the protein levels of activated CASP1 and GSDMD-N (Fig. 2A, B), and inhibited IL1B production (Fig. 2A–C) in β-GP treated VSMCs. Moreover, cell lysis and pyroptotic cell death were reversed by VX-765, as demonstrated by the reduction in LDH release (Fig. 2D) and percentage of PI-positive cells (Fig. 2E). Importantly, VX-765 effectively reduced calcium deposition in VSMCs (Fig. 2F, G) and mouse aortic rings (Fig. 2H, I) after β-GP treatment for 7 days. Therefore, these data suggested that CASP1-dependent pyroptotic cell death occurred in β-GP-treated VSMCs and played an important role in VSMC calcification.

### β-GP-induced pyroptotic cell death depends on ROS/NLRP3 inflammasome activation in VSMCs

**Fig. 3:**
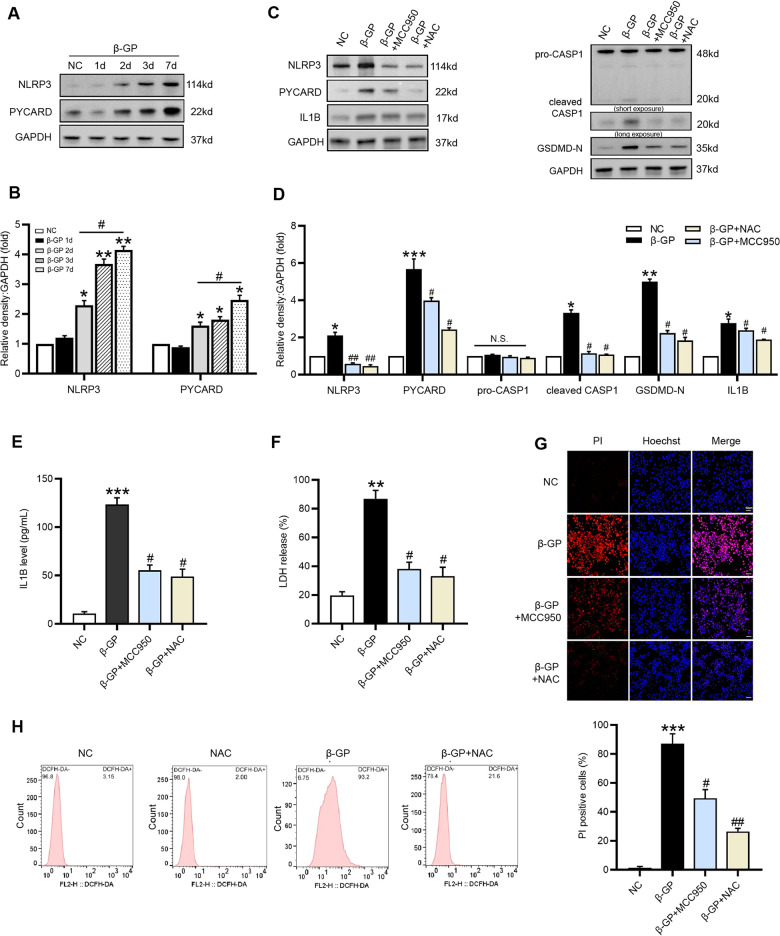
ROS-mediated NLRP3 inflammasome activation is involved in β-GP-induced VSMC pyroptosis. VSMCs were treated with β-GP (10 mM) for the indicated time. **A** The protein levels of NLRP3 and PYCARD were determined by western blotting. **B** Quantification of the results shown in **A**. The data are presented as means ± SEM. *P < 0.05, **P < 0.01 vs. control group; ^*#*^*P* < 0.05, ^*##*^*P* < 0.01 vs^.^ indicated group. VSMCs were incubated in the presence of β‐GP (10 mM) medium with the ROS inhibitor NAC (20 μM) or NLRP3 inhibitor MCC950 (100 μM) as indicated. **C** The protein levels of pyroptosis-related markers were determined by western blotting. **D** Quantification of the results shown in **C**. **E** The IL1B content in VSMC culture supernatants was determined by ELISA. **F** The release of LDH was detected using the LDH Assay Kit. **G** The percentage of PI-positive cells was measured using Hoechst 33342 (blue)/PI (red) double staining (top: Representative images; bottom: Quantitative analysis of PI-positive cells). Scale bar = 50 μm. **H** The intracellular ROS level was detected using the fluorescent probe DCFH-DA and measured by flow cytometry. Data are expressed as mean ± SEM. *P < 0.05, **P < 0.01, ***P < 0.001 vs. control group; ^*#*^*P* < 0.05, ^*##*^*P* < 0.01 vs. β-GP group; and N.S. not significant.

The NLRP3 inflammasomes contains NLRP3, the adaptor PYD and CARD domain-containing (PYCARD/ASC) and the effector pro-CASP1. NLRP3 can recruit PYCARD and cleave pro-CASP1 into its active forms [5]. Results showed that β-GP significantly induced the protein expression of NLRP3 and PYCARD as early as Day 2 and persisted for 7 days in VSMCs (Fig. 3A, B). Subsequently, we performed NLRP3 inhibitory experiments using the NLRP3 inhibitor MCC950. MCC950 addition resulted in a significant reduction in the protein level of NLRP3 in calcified VSMCs (Fig. 3C, D). Moreover, NLRP3 silencing notably reversed the upregulation of PYCARD, cleaved CASP1, and GSDMD-N (Fig. 3C, D), and production of IL1B (Fig. 3C–E). Similarly, NLRP3 silencing also suppressed LDH release (Fig. 3F) and the percentage of PI-positive cells (Fig. 3G), indicating that β-GP-induced pyroptotic cell death in VSMCs was mediated by NLRP3 inflammasome activation. In addition, our results demonstrated that β-GP treatment resulted in the accumulation of intracellular ROS in VSMCs, an effect that was abrogated by NAC, a specific ROS inhibitor (Fig. 3H). Importantly, we observed that NAC markedly inhibited β-GP-induced protein expression of NLRP3 inflammasome components and pyroptosis-related markers (Fig. 3C–E). Furthermore, NAC treatment reduced LDH release (Fig. 3F) and PI-positive cells (Fig. 3G) in β-GP-treated VSMCs. The above findings suggested the importance of ROS in β-GP-induced NLRP3 inflammasome activation and pyroptosis in VSMCs.

### Irisin attenuates β-GP-induced VSMC calcification by suppressing the NLRP3-mediated pyroptotic cell death pathway

To determine whether Irisin affects VSMC calcification, VSMCs were treated with β-GP in the presence or absence of Irisin (50, 100 ng/ml) for 7 days. Fig. S1A and B showed that exposure of VSMCs to Irisin dose-dependently decreased β-GP-induced calcium deposition. Moreover, Irisin (100 ng/ml) markedly inhibited β-GP-induced the protein expression levels of NLRP3 inflammasome components and pyroptosis-related markers (Fig. S1C–E). And membrane rupture (Fig. S1F, G) and DNA fragmentation (Fig. S1H) were markedly alleviated after applying 100 ng/ml Irisin. Therefore, Irisin (100 ng/ml) was used as an effective concentration for further in vitro experiments.

**Fig. 4:**
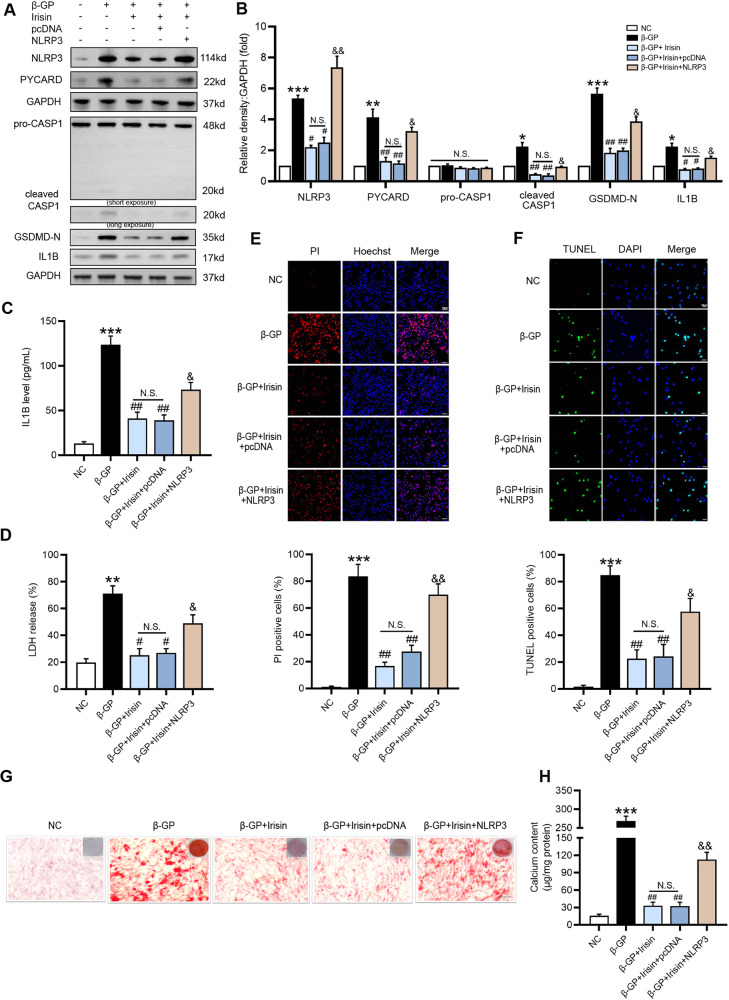
Overexpression of *Nlrp3* attenuates the inhibitory effect of Irisin on β-GP-induced VSMC pyroptosis and calcification. VSMCs were transfected with the pcDNA empty vector (pcDNA) or *Nlrp3*-expressed plasmid (NLRP3) for 48 h, and were subsequently treated with β-GP (10 mM) in the presence or absence of 100 ng/ml of Irisin for 7 days. **A** The protein levels of pyroptosis-related markers were determined by western blotting. **B** Quantification of the results shown in A. **C** The IL1B content in VSMC culture supernatants was determined by ELISA. **D** The release of LDH was detected using the LDH Assay Kit. **E** The percentage of PI-positive cells was measured using Hoechst 33342 (blue)/PI (red) double staining (top: Representative images; bottom: Quantitative analysis of PI-positive cells). **F** The percentage of TUNEL-positive cells (green) was evaluated by TUNEL staining (top: Representative images; bottom: Quantitative analysis of TUNEL-positive cells). Scale bar = 50 μm for (**E**); Scale bar = 20 μm for (**F**). **G** Calcium deposition in VSMCs was assessed by Alizarin red staining (positive staining: red; scale bar=100 μm). **H** Quantitative analysis of calcium deposition in VSMCs normalized to the protein content. Data are expressed as mean ± SEM. *P < 0.05, **P < 0.01, ***P < 0.001 vs. control group; ^*#*^*P* < 0.05, ^*##*^*P* < 0.01 vs. β-GP group;^*&*^*P* < 0.05, ^*&&*^*P* < 0.01 vs. β-GP + Irisin+pcDNA group; and N.S. not significant.

Next, we overexpressed *Nlrp3* in VSMCs and performed the following studies. The *Nlrp3* mRNA (Fig. S2A) and protein (Fig. S2B) expression levels were significantly increased in VSMCs that were transfected with the *Nlrp3* expression plasmids compared with the control group. Although Irisin suppressed the protein expression levels of NLRP3 inflammasome components and pyroptosis-related markers, which were induced by β-GP, overexpression of *Nlrp3* could reverse the Irisin-mediated preventive effects (Fig. 4A–C). Most importantly, overexpression of *Nlrp3* significantly attenuated the inhibitory effect of Irisin on pyroptotic cell death (Fig. 4D–F), resuming β-GP-induced calcification in VSMCs (Fig. 4G, H). These results indicated that Irisin protected VSMCs against calcification by inhibiting the NLRP3-mediated pyroptosis pathway.

### Irisin induces autophagy and enhances autophagic flux in VSMCs treated with β-GP

**Fig. 5:**
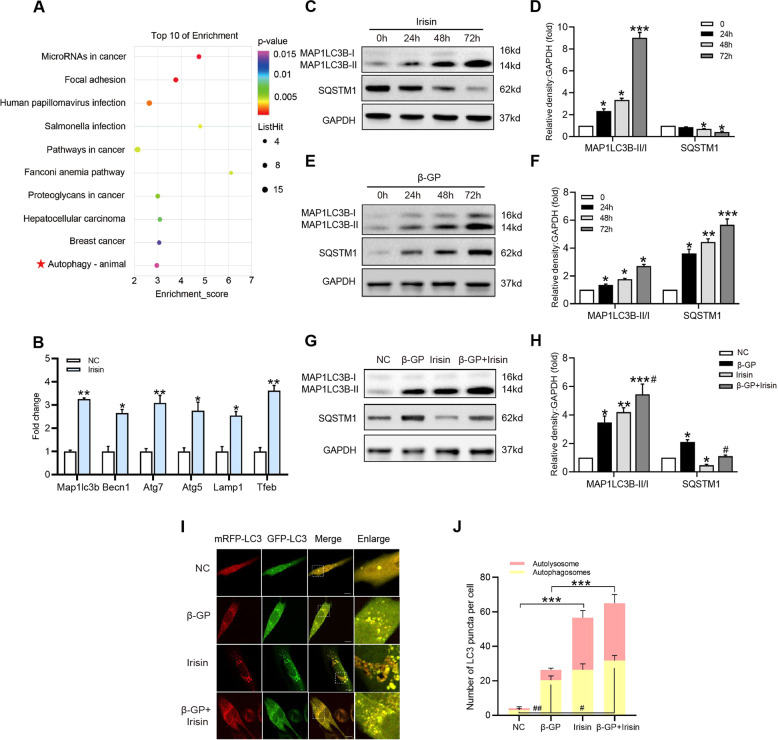
Irisin treatment induces autophagy and restores autophagic flux in calcified VSMCs. **A** Pathway analysis of the 277 target genes based on KEGG database. The top 10 positively enriched pathways are shown in the bubble chart. The x-axis represents the enrichment score, y-axis represents the enriched pathways. The color of the bubble represents the *P* value of the pathway enrichment. The size of each bubble represents the number of different genes contained in the pathway. **B** VSMCs were treated with Irisin (100 ng/ml) for 72 h and the mRNA level of *Map1lc3b*, *Becn1*, *Atg7*, *Atg5*, *Lamp1*, and *Tfeb* was assessed by qRT-PCR. Data are expressed as mean ± SEM. *P < 0.05, **P < 0.01 vs. control group. **C–F** VSMCs were treated with Irisin (100 ng/ml) or β-GP (10 mM) for the indicated time. Protein levels of MAP1LC3B and SQSTM1were determined by western blotting. **G, H** VSMCs were incubated with or without Irisin (100 ng/ml) in the presence of β-GP (10 mM) for 72 h. Protein levels of MAP1LC3B and SQSTM1 were evaluated by western blotting. Data are expressed as mean ± SEM. *P < 0.05, **P < 0.01, ***P < 0.001 vs corresponding control group; ^*#*^*P* < 0.05, ^*##*^*P* < 0.01 vs corresponding β-GP group. **I** Confocal microscopy observation of mRFP-GFP-LC3 adenovirus transfected VSMCs treated as indicated (Magnification: ×63, Scale bars = 10 μm). **J** Autophagosome (yellow dots) and autolysosome (red dots) numbers in each group were calculated. Data are expressed as mean ± SEM. *P < 0.05, **P < 0.01, ***P < 0.001 vs. indicated group; ^*#*^*P* < 0.05, ^*##*^*P* < 0.01 vs. indicated group.

To further explore the mechanism by which Irisin inhibits VSMC pyroptosis and calcification, we used RNA sequencing (RNA-seq) to analyze the differentially expressed genes in β-GP treated, and Irisin and β-GP co-treated VSMCs, and used un-treated VSMCs as controls (data not shown). Moreover, we found that 277 gene expression levels were upregulated or downregulated in Irisin and β-GP co-treated VSMCs compared with those in β-GP treated VSMCs (*P* < 0.05). Next, we performed functional annotation on these genes using the Kyoto Encyclopedia of Genes and Genomes (KEGG) Database platforms. The top 10 enriched KEGG pathways are presented (Fig. 5A). Studies have revealed that autophagy could exert its inhibitory effect on cell pyroptosis and inflammatory cytokine release through elimination of inflammasomes and other essential pyroptotic components [13, 14, 33]. Therefore, we focused on the autophagy pathway. Fig. 5B showed that Irisin treatment markedly increased the mRNA expression levels of the major autophagy-related genes (*Map1lc3b*, *Becn1*, *Atg7*, *Atg5*, *Lamp1*, and *Tfeb*) in VSMCs. Western blot analysis showed a time-dependent increase in the MAP1LC3B-II generation and SQSTM1 degradation (autophagy markers) in Irisin-treated VSMCs, suggesting that it is a potent autophagy inducer (Fig. 5C, D).

Subsequently, we observed the effect of Irisin on the autophagy levels in calcified VSMCs. Consistent with previous studies [21], we showed that the MAP1LC3B-II/I ratio and SQSTM1 level were elevated at 24 h and significantly increased at 48 h and 72 h, indicating the inhibition of autophagic flux in calcified VSMCs (Fig. 5E, F). Importantly, after Irisin treatment for 72 h, the MAP1LC3B-II/I ratio (Fig. 5G, H) was further enhanced, and SQSTM1 accumulation (Fig. 5G, H) was significantly alleviated, indicating that Irisin activates autophagy and autophagic flux in calcified VSMCs. This finding was further supported by autophagy flux analysis. Compared with the control group, Irisin treatment alone markedly induced an increase in both autophagosomes and autolysosomes. However, increased autophagosomes instead of autolysosomes were observed in β-GP-treated cells, suggesting that the maturation procession from autophagosomes to autolysosomes were blocked. Importantly, in the Irisin and β-GP co-treated cells, more autophagosomes and autolysosomes were detected than those in the β-GP treated cells (Fig. 5I, J). These results indicated that Irisin induced autophagy activation and restored defective autophagic flux during VSMC calcification.

### Blocking autophagic flux reverses the protective effect of Irisin against β-GP- induced VSMC pyroptosis and calcification

**Fig. 6:**
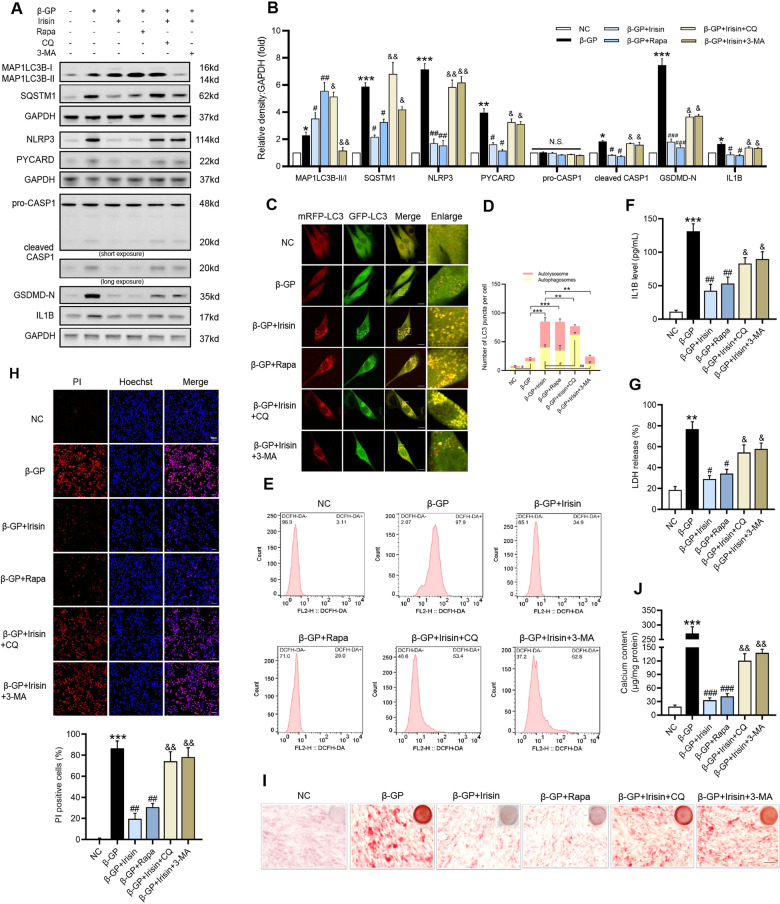
Irisin protects VSMCs from β-GP-induced pyroptotic cell death and calcification by promoting autophagic flux. VSMCs were cultured in the presence of β-GP (10 mM) with or without of Irisin, and were subsequently treated with rapamycin (Rapa, 200 nM), 3-methyladenine (3-MA, 5 mM) or chloroquine (CQ,25 µM) for 72 h (autophagy) or 7 days (calcification). **A** The protein levels of autophagy- and pyroptosis-related markers were determined by western blotting. **B** Quantification of the results shown in A. **C** Confocal microscopy observation of mRFP-GFP-LC3 adenovirus transfected VSMCs treated as indicated (Magnification: ×63, Scale bars=10 μm). **D** Autophagosome (yellow dots) and autolysosome (red dots) numbers in each group were calculated. Data are expressed as mean ± SEM. *P < 0.05, **P < 0.01, ***P < 0.001 vs. indicated group; ^*#*^*P* < 0.05, ^*##*^*P* < 0.01 vs. indicated group. **E** The intracellular ROS level was detected using the fluorescent probe DCFH-DA and measured by flow cytometry. **F** The IL1B content in VSMC culture supernatants was determined by ELISA. **G** The release of LDH was detected using the LDH Assay Kit. **H** The percentage of PI-positive cells was measured using Hoechst 33342 (blue)/PI (red) double staining (top: Representative images; bottom: Quantitative analysis of PI-positive cells). Scale bar = 50 μm. **I** Calcium deposition in VSMCs was assessed by Alizarin red staining. Scale bar = 100 μm. **J** Quantitative analysis of calcium deposition in VSMCs normalized to the protein content. Data are expressed as mean ± SEM. *P < 0.05, **P < 0.01, ***P < 0.001 vs. control group; ^*#*^*P* < 0.05, ^*##*^*P* < 0.01, ^*###*^*P* < 0.001 vs. corresponding β-GP group; ^&^*P* < 0.05, ^&&^*P* < 0.01 vs. corresponding β-GP + Irisin group; and N.S. not significant.

Based on the above results, we further investigated the role of autophagy in Irisin-mediated pyroptosis inhibition during VSMC calcification. Rapamycin was used to enhance autophagic flux in β-GP-treated VSMCs. The inhibition effects of CQ and 3-MA on autophagy and autophagic flux were testified in calcified VSMCs (Fig. 6A–D). When Irisin was added to the medium of β-GP-treated VSMCs, we found that β-GP-induced ROS production was suppressed. This result was consistent with that obtained when rapamycin was added to the medium of β-GP-treated VSMCs. However, the preventive effects of Irisin on β-GP-induced excessive ROS production were blunted by CQ or 3-MA (Fig. 6E). Moreover, Irisin treatment ameliorated β-GP-induced NLRP3 inflammasome activation and pyroptosis levels in VSMCs, as reflected by decreased protein expression of NLRP3 inflammasome components and pyroptosis markers (Fig. 6A, B), IL1B production (Fig. 6F), LDH release (Fig. 6G), and PI-positive cells (Fig. 6H). However, the inclusion of CQ or 3-MA reversed the Irisin-mediated amelioration (Fig. 6A, B, F–H). Similar to treatment with the rapamycin, Irisin inhibited β-GP-induced VSMC calcification, whereas both CQ and 3-MA alleviated the inhibitory effect of Irisin (Fig. 6I, J). These results demonstrated that Irisin inhibited β-GP-induced ROS production, NLRP3 inflammasome activation, pyroptosis, and calcium deposition by facilitating autophagic flux in VSMCs.

### Irisin treatment protects against medial calcification in vivo and ex vivo

**Fig. 7:**
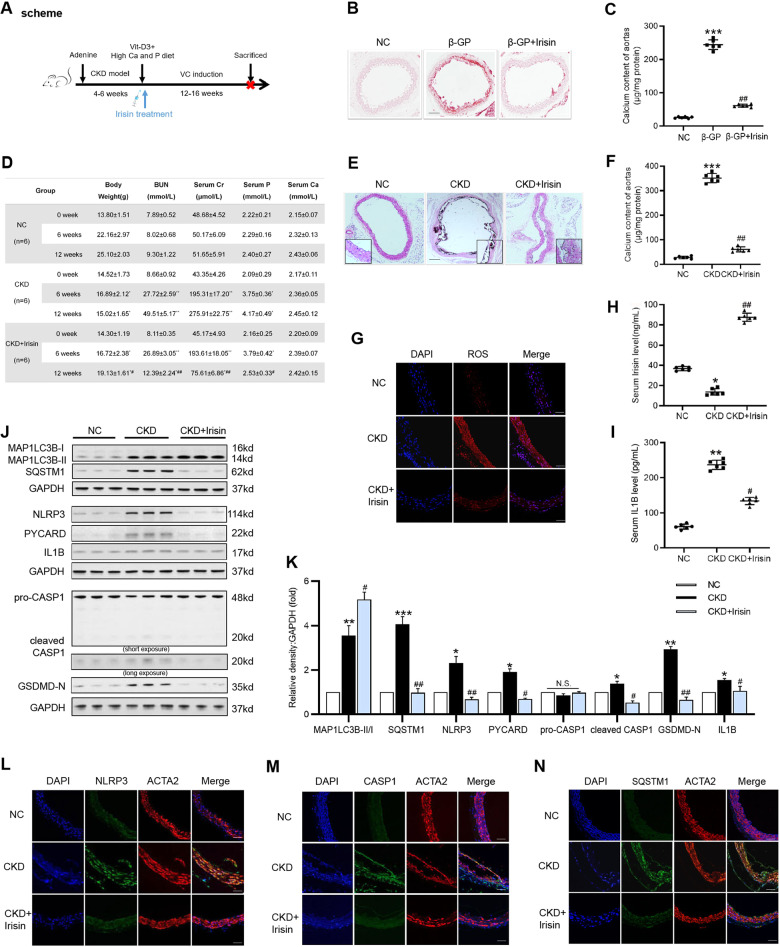
Irisin prevents medical VC ex vivo and in vivo. **A** Scheme of the construction of the CKD-associated VC animal model. **B-C** Mouse aortic rings were treated with Irisin in medium containing β-GP (10 mM) for 7 days. Calcification was assessed by Alizarin red staining (**B**), and the calcium content in the aortas was quantified (**C**). (positive staining: red; scale bar=100 μm). *P < 0.05, **P < 0.01, ***P < 0.001 vs. control group; ^*#*^*P* < 0.05, ^*##*^*P* < 0.01 vs. β-GP group. **D** Mice serum levels of blood urea nitrogen (BUN), creatinine (Cr), calcium (Ca), and phosphate (P) were measured. Values are expressed as mean ± SEM (*n* = 6 per group). *P < 0.05, **P < 0.01 vs. the age-marched controls. ^*#*^*P* < 0.05, ^*##*^*P* < 0.01 vs. the age-marched CKD group. CKD mice were injected subcutaneously with vitamin D3(Vit-D3), fed a high Ca and P diet, and treated with Irisin or normal saline (NS) for 12 weeks. Normal mice with the same age were used as controls. **E** Calcium deposition in the abdominal aortas was detected by Von Kossa staining (**D**). (positive staining: brown to black; scale bar = 100 μm). **F** The calcium content in the abdominal aortas was quantified. **G** The ROS level in aortic tissues was detected using the fluorescent probe DCFH-DA and visualized using confocal microscopy. (shown in red, Magnification: ×400, scale bar = 100 μm). Cell nuclei were visualized using DAPI (blue). **H** Serum Irisin level was evaluated by ELISA. **I** Serum IL1B level was evaluated by ELISA. **J** The protein levels of autophagy- and pyroptosis-related markers were determined by western blotting. **K** Quantification of the results shown in **J**. Representative immunofluorescent staining images for NLRP3 (green, **L**), CASP1 (green, **M**), and SQSTM1 (green, **N**). The sections were co-stained with ACTA2/α-SMA to outline the VSMC area. (Magnification: ×400, scale bar=100 μm). Data are expressed as mean ± SEM. *P < 0.05, **P < 0.01, ***P < 0.001 vs. control group; ^*#*^*P* < 0.05, ^*##*^*P* < 0.01 vs. CKD group. Calcium: Ca, phosphate: P.

To examine the effect of Irisin on VC ex vivo, mouse abdominal aortas were cut into rings and cultured in medium containing β-GP for 7 days. Results showed that Irisin significantly inhibited β-GP-induced calcium deposition in mouse aortic rings (Fig. 7B, C). Subsequently, we employed an adenine-induced mouse CKD model to assess the therapeutic effect of Irisin on arterial calcification in vivo. After 6 weeks of an adenine diet, the CKD mice showed severe renal failure, which was confirmed by the increased levels of BUN and Cr (Fig. 7D). Next, CKD mice were injected subcutaneously with vitamin D3 and fed a high calcium and phosphate diet to establish a VC model (Fig. 7A). At the end of the experiment, we found that Irisin treatment effectively reduced aortic calcification (Fig. 7E, F) and ROS production (Fig. 7G) in CKD mice. Moreover, serum levels of Irisin in the CKD group decreased compared with that in the control group, while the serum levels of Irisin significantly enhanced in the Irisin treatment group (Fig. 7H). Western blot analysis showed that Irisin treatment increased the ratio of MAP1LC3B-II/I and SQSTM1 degradation and decreased the expression of NLRP3 inflammasome components and pyroptosis-related markers compared with that in the CKD group (Fig. 7J, K). And immunofluorescence staining results for NLRP3 (Fig. 7L), CASP1 (Fig. 7M), and SQSTM1 (Fig. 7N) were consistent with the western blot results. In addition, ELISA showed that Irisin treatment significantly decreased the serum levels of IL1B in CKD mice (Fig. 7I). These results suggested that Irisin could enhance autophagy, inhibit ROS production and VSMC pyroptosis, and ameliorate the progression of VC in CKD mice.

## Discussion

**Fig. 8:**
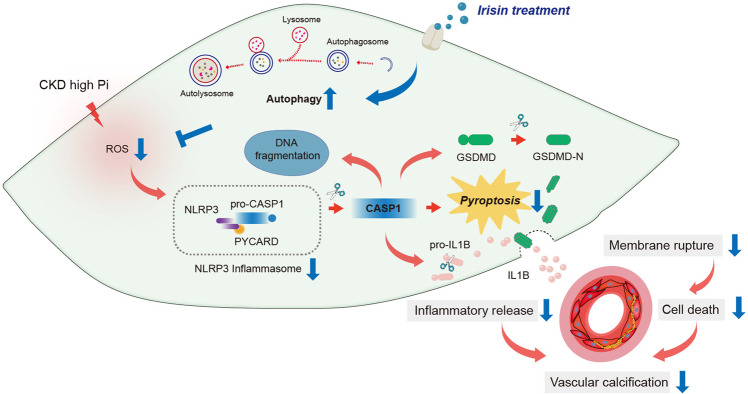
A graphic illustration of the protective role of Irisin in CKD-associated VC. High phosphate (Pi) induces the production of ROS, which activates NLRP3 inflammasome, leads to the activation of CASP1-dependent pyroptotic cell death, and release of inflammatory factors in VSMCs, and ultimately promotes the formation of VC in CKD (**Red arrows**). Irisin induces autophagy and reduces ROS production. These effects lead to inhibition of NLRP3-mediated pyroptotic cell death, proinflammatory response, and calcium deposition in VSMCs, and ameliorate the progression of VC in CKD (**Blue arrows**).

This study is the first to show that VSMC pyroptosis is a crucial event in the pathogenesis of VC in CKD. Moreover, our findings clearly indicate that Irisin plays a protective role against pyroptotic cell death and calcification in the aortas of CKD mice and β-GP-treated VSMCs. More importantly, mechanistic studies reveal that Irisin enhances autophagy and reduces ROS production in VSMCs; this effect leads to the inhibition of NLRP3-mediated pyroptotic cell death and the pro-inflammatory response, and eventually ameliorates VSMCs calcification (Fig. 8).

Medial VC is a common complication of CKD patients, and the development of VC during CKD is a highly regulated process which involves complex interactions of multiple factors [2, 34]. Dysregulation of VSMCs plays an essential role in VC formation. Interaction between VSMC death and inflammation leads to the progression of VC [2, 35, 36]. Hence, discovering new modulatory mechanisms that control both VSMC death and inflammation is necessary to prevent VC progression in CKD.

Pyroptosis is a pro-inflammatory form of cell death that primarily depends on CASP1 activation. CASP1-dependent pyroptosis plays a pivotal role in the pathogenesis of cardiovascular disease and can be observed in endothelial cells [37, 38], macrophages [39], and so on. Recent findings have revealed that CASP1 is activated in high phosphate-induced VSMC calcification [10, 11]. However, whether pyroptotic cell death induced by activated CASP1 is involved in VSMC calcification has never been reported. In the present study, we induced VSMC calcification by treating VSMCs with β-GP for 7 days. During this process, VSMCs displayed features of pyroptosis, as evidenced by the upregulated expression of pyroptosis-related proteins (cleaved CASP1, GSDMD-N, IL1B) and pyroptotic cell death (increased numbers of PI-positive cells and LDH release). Importantly, the CASP1 inhibitor VX-765 ameliorated the CASP1 activation, and subsequently inhibited pyroptotic cell death and calcium deposition in β-GP-treated VSMCs, indicating that pyroptotic cell death occurred in calcifying VSMCs and might be a crucial mechanism for VSMC calcification.

CASP1 dependent pyroptosis is activated by the assembly and activation of inflammasomes. NOD-like receptors (NLRP1, NLRP3, NLRP6, NLRP9, and NLRC4), pyrin, and absent in melanoma 2 (AIM2) are the major members of the inflammasome family. Among them, NLRP3 inflammasome plays an important role in CKD-associated VC [10, 11, 40, 41]. Consistently, our results showed that the expression of NLRP3 and PYCARD was upregulated in calcified VSMCs. Furthermore, silencing NLRP3 remarkably decreased the levels of pyroptosis-related markers and inhibited pyroptotic cell death in VSMCs treated with β-GP. These results suggested that NLRP3 inflammasome activation was required for β-GP -induced VSMC pyroptosis. Multiple upstream mechanisms which can induce the activation of NLRP3 inflammasome have been identified, including ROS generation [37], cathepsin B leakage from lysosomes [42], mitochondrial dysfunction [43], and potassium efflux [44]. Among these stimuli, ROS generation is the critical signaling for NLRP3 inflammasome activation [45]. Numerous studies have demonstrated that VC is associated with ROS production, and excessive ROS contribute to the activation of signaling pathways related to inflammation and cell death, and thereby accelerate the development of VC [2, 46–48]. In the present study, we found that β-GP could induce ROS generation and accumulation in VSMCs, inhibition of intracellular ROS production significantly ameliorated inflammasome activation and pyroptotic cell death in calcified VSMCs. Together, these results indicated that ROS generation was necessary for NLRP3 inflammasome activation and pyroptosis during VSMC calcification. Targeting the ROS/NLRP3-mediated pyroptosis pathway may serve as a new therapeutic target for VC.

Recently, Irisin has gained increased attention for its protective effects in human metabolic diseases. This myokine is a cleaved product from its precursor fibronectin type III domain-containing protein 5 (FNDC5) and is shed into the extracellular milieu and circulation [16, 49, 50]. The serum Irisin levels are significantly lower in patients with cardiovascular diseases, particularly atherosclerosis. Irisin treatment reduced the degree of aortic atherosclerotic plaques in *Apolipoprotein E*-deficient mice [32, 51]. Our study recently reported that the plasma levels of Irisin are lower in uremic patients with VC than in those without VC [18, 19]. Moreover, lower Irisin levels are associated with increased cardiovascular and cerebrovascular disease mortality in CKD patients [19]. In the present study, our results from in vitro, ex vivo and in vivo experiments indicated that Irisin has an inhibitory effect on VC. A recent study demonstrated that Irisin ameliorated pressure overload-induced cardiac hypertrophy by inhibiting pyroptosis of cardiomyocytes [52]. Most recently, Li et al. [53] revealed the role of Irisin as a promising anti-pyroptosis agent in inhibiting the progression of septic liver injury. Based on these findings, we found that Irisin repressed β-GP-induced NLRP3 inflammation activation and pyroptotic cell death in VSMCs, and *Nlrp3* overexpression reduced the protective effect of Irisin. These results further confirmed that Irisin protected VSMCs against calcification by inhibiting the NLRP3-mediated pyroptosis pathway.

Autophagy is a critical biological response to maintain cell homeostasis and plays a protective role in CKD-associated VC [2, 21, 48, 54, 55]. Increasing evidence has revealed that autophagy could reduce oxidative damage and ROS levels through removal of protein aggregates and damaged organelles such as mitochondria [12, 13, 56]. Recent studies have demonstrated an association between Irisin and autophagy in different tissues and diseases [17, 57–59]. Our present study demonstrated for the first time that Irisin significantly induced autophagy and promoted autophagic flux in β-GP-treated VSMCs and in the aortas of CKD mice. Moreover, autophagy induction by rapamycin markedly reduced the excessive ROS production induced by β-GP in VSMCs. And the beneficial effects of Irisin were consistent with those produced by rapamycin. However, inhibition of autophagy markedly diminished the ability of Irisin to protect VSMCs against β-GP-induced ROS production, pyroptotic cell death, and calcification. These findings suggested that the inhibitory effects of Irisin on β-GP-induced VSMC calcification are partly attributed to the enhanced autophagy, which can reduce ROS production and inhibit NLRP3-mediated pyroptotic cell death.

In accordance with the in vitro results, exogenous Irisin treatment could promote autophagy and reduce ROS accumulation and the activation of NLRP3-mediated pyroptosis signaling in the aortas of CKD mice. Moreover, Irisin treatment markedly inhibited the formation of VC in CKD mice. In addition, we found that irisin treatment lowered the levels of serum creatinine and BUN in the CKD mice. These results were consistent with recent studies [60, 61] and suggested that the important role of Irisin in attenuating the progression of kidney damage and improving renal function in CKD. Importantly, serum phosphate levels were markedly reduced in the CKD mice after Irisin treatment, which might be another important mechanism of the Irisin-mediated inhibitory effect on CKD -associated VC.

Some limitations must be noted in this study. First, our study mainly focused on the effects of Irisin on autophagy activity in the VC of CKD, and the signaling pathways for Irisin-induced autophagy in CKD-associated VC require further exploration according to the RNA-Seq results. Second, the present study suggested that Irisin might have a direct therapeutic effect on VC in CKD mice. In future studies, we will explore the role of its precursor, the FNDC5 gene, in VC under CKD conditions both in vitro and in vivo. Therefore, further studies are warranted.

In conclusion, our study demonstrates that pyroptosis plays an essential role in the pathogenesis of VC in CKD, and Irisin ameliorates the development of VC by promoting autophagy, reducing oxidative stress, and suppressing NLRP3-dependent pyroptosis pathway. These findings indicate that the inhibition of pyroptosis or clinical application of Irisin may be a potential therapeutic strategy to prevent and treat VC in CKD patients.

## Supplementary information

Supplementary Table

Supplementary Figure 1

Supplementary Figure 2

Supplementary Figure 3

Supplementary Figure legends

checklist-CDDIS-21-3630RR

## Data Availability

All data generated or analyzed during this study are available from the corresponding author on reasonable request.
