# Supplementary material for: Irisin protects against vascular calcification by activating autophagy and inhibiting NLRP3-mediated vascular smooth muscle cell pyroptosis in chronic kidney disease
Source: Cell Death Dis. 2022 Mar 30;13(3):283. doi: 10.1038/s41419-022-04735-7 (PMC8967887; doi:10.1038/s41419-022-04735-7)
Supplement: Supplementary file 1 — Supplementary Table [file 41419_2022_4735_MOESM1_ESM.docx]

**Table S1 Sequences of primers used for qRT-PCR**

The primer sequences contained in this study were as follows (<http://pga.mgh.harvard.edu/primerbank/>):

| **Gene** | **Forward primer (5’-3’)** | **Reverse primer (5’-3’)** |
| --- | --- | --- |
| *Map1lc3b* | CGCTTGCAGCTCAATGCTAAC | CTCGTACACTTCGGAGATGGG |
| *Becn1* | AGGCGAAACCAGGAGAGAC | CCTCCCCGATCAGAGTGAA |
| *Atg7* | TCTGGGAAGCCATAAAGTCAGG | GCGAAGGTCAGGAGCAGAA |
| *Atg5* | TGTGCTTCGAGATGTGTGGTT | GTCAAATAGCTGACTCTTGGCAA |
| *Lamp1* | TGCTCCGGGATGCCACTAT | TGTTGTCCTTTTTCAGGTAGGTG |
| *Tfeb* | AAGGTTCGGGAGTATCTGTCTG | GGGTTGGAGCTGATATGTAGCA |
| *Gapdh* | TGACCTCAACTACATGGTCTACA | CTTCCCATTCTCGGCCTTG |
