# Supplementary figures and images for: Irisin protects against vascular calcification by activating autophagy and inhibiting NLRP3-mediated vascular smooth muscle cell pyroptosis in chronic kidney disease
Source: Cell Death Dis. 2022 Mar 30;13(3):283. doi: 10.1038/s41419-022-04735-7 (PMC8967887; doi:10.1038/s41419-022-04735-7)

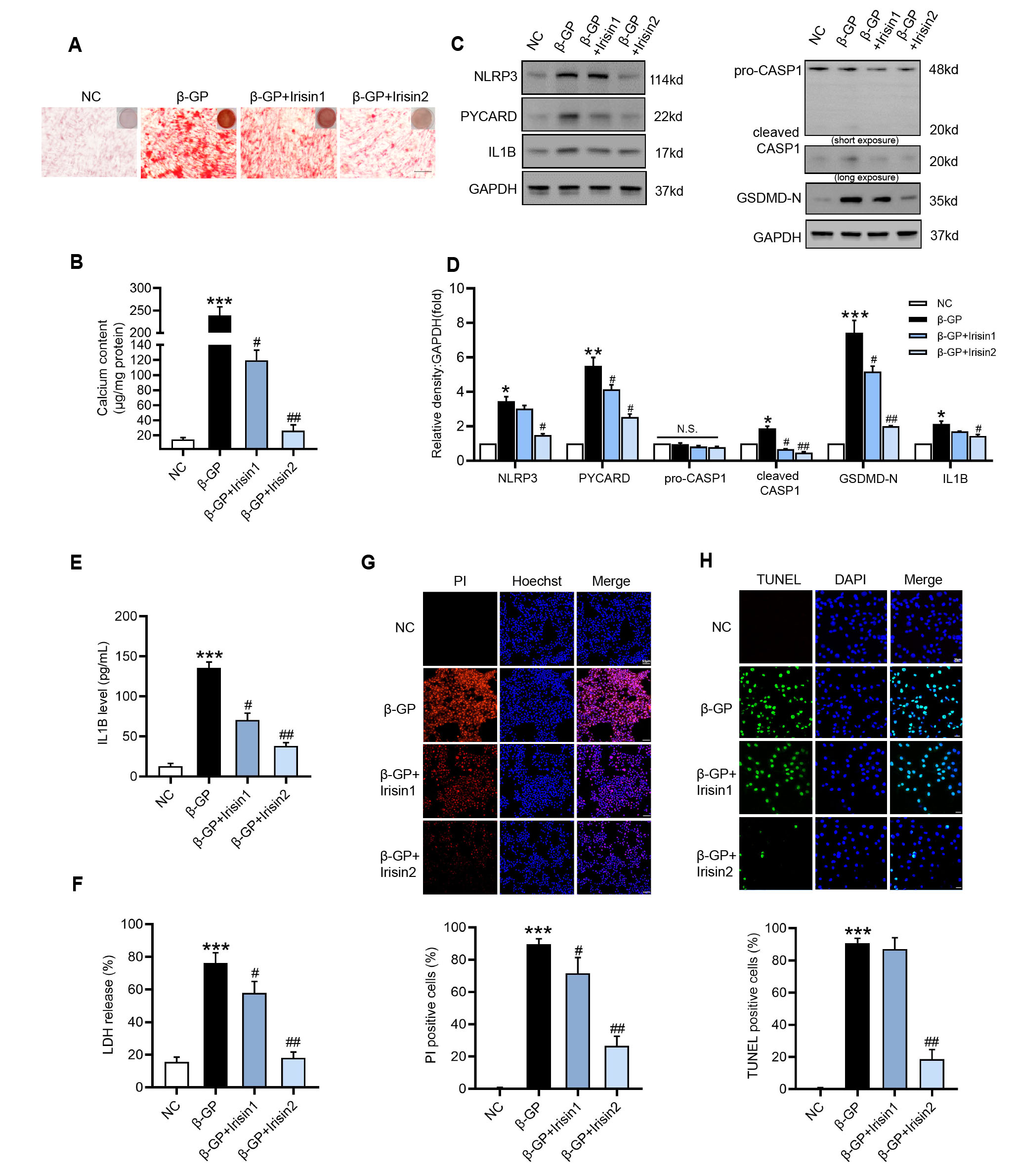

Supplement: Supplementary file 2 — Supplementary Figure 1 [file 41419_2022_4735_MOESM2_ESM.jpg]

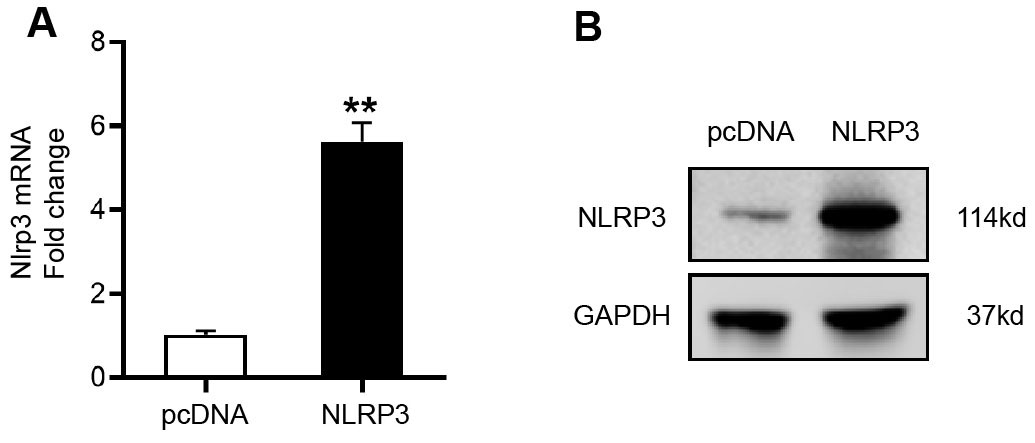

Supplement: Supplementary file 3 — Supplementary Figure 2 [file 41419_2022_4735_MOESM3_ESM.jpg]
