# Supplementary material for: Irisin protects against vascular calcification by activating autophagy and inhibiting NLRP3-mediated vascular smooth muscle cell pyroptosis in chronic kidney disease
Source: Cell Death Dis. 2022 Mar 30;13(3):283. doi: 10.1038/s41419-022-04735-7 (PMC8967887; doi:10.1038/s41419-022-04735-7)
Supplement: Supplementary file 4 — Supplementary Figure 3 [file 41419_2022_4735_MOESM4_ESM.pdf]

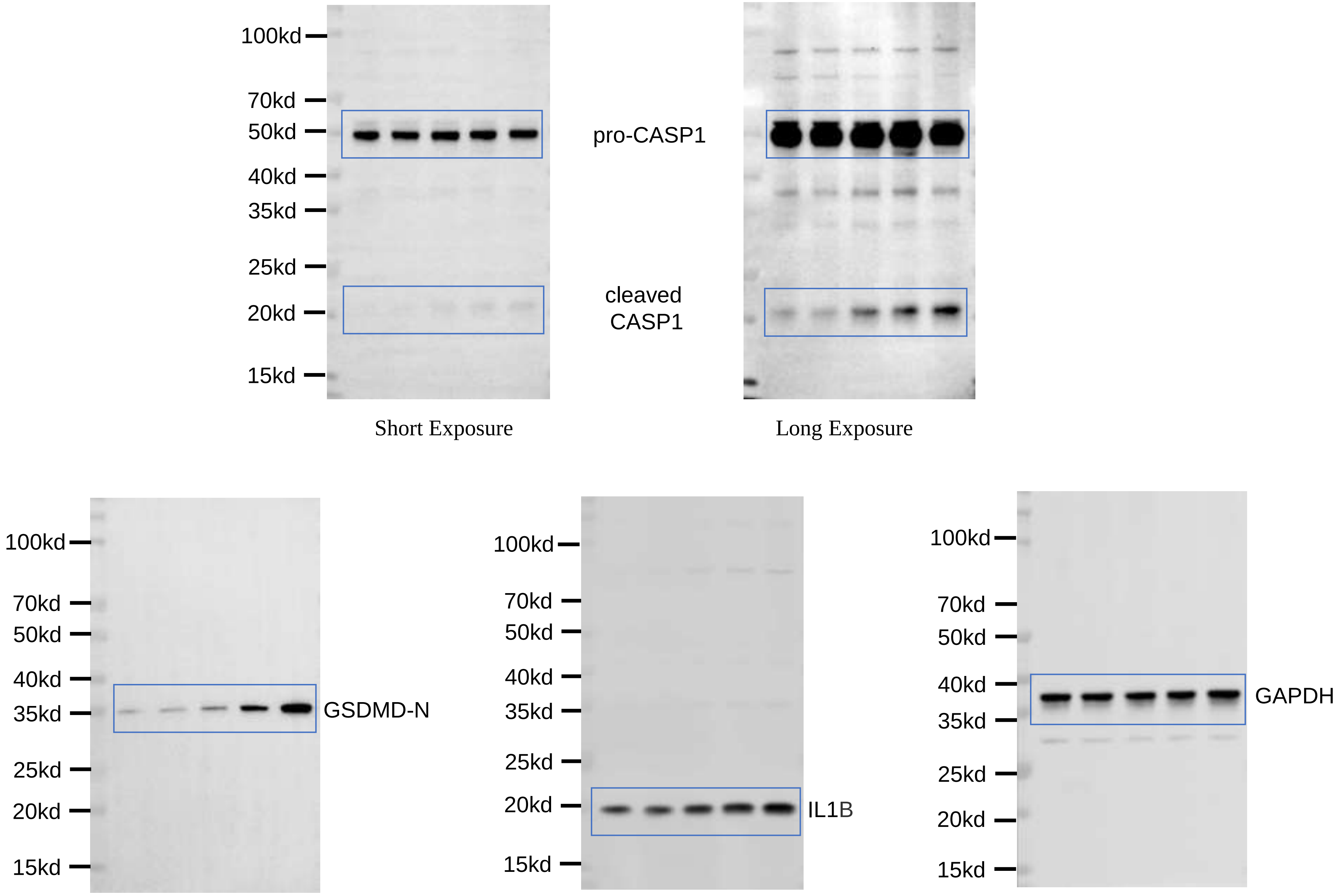

Uncropped versions of blots shown in Figure 1C

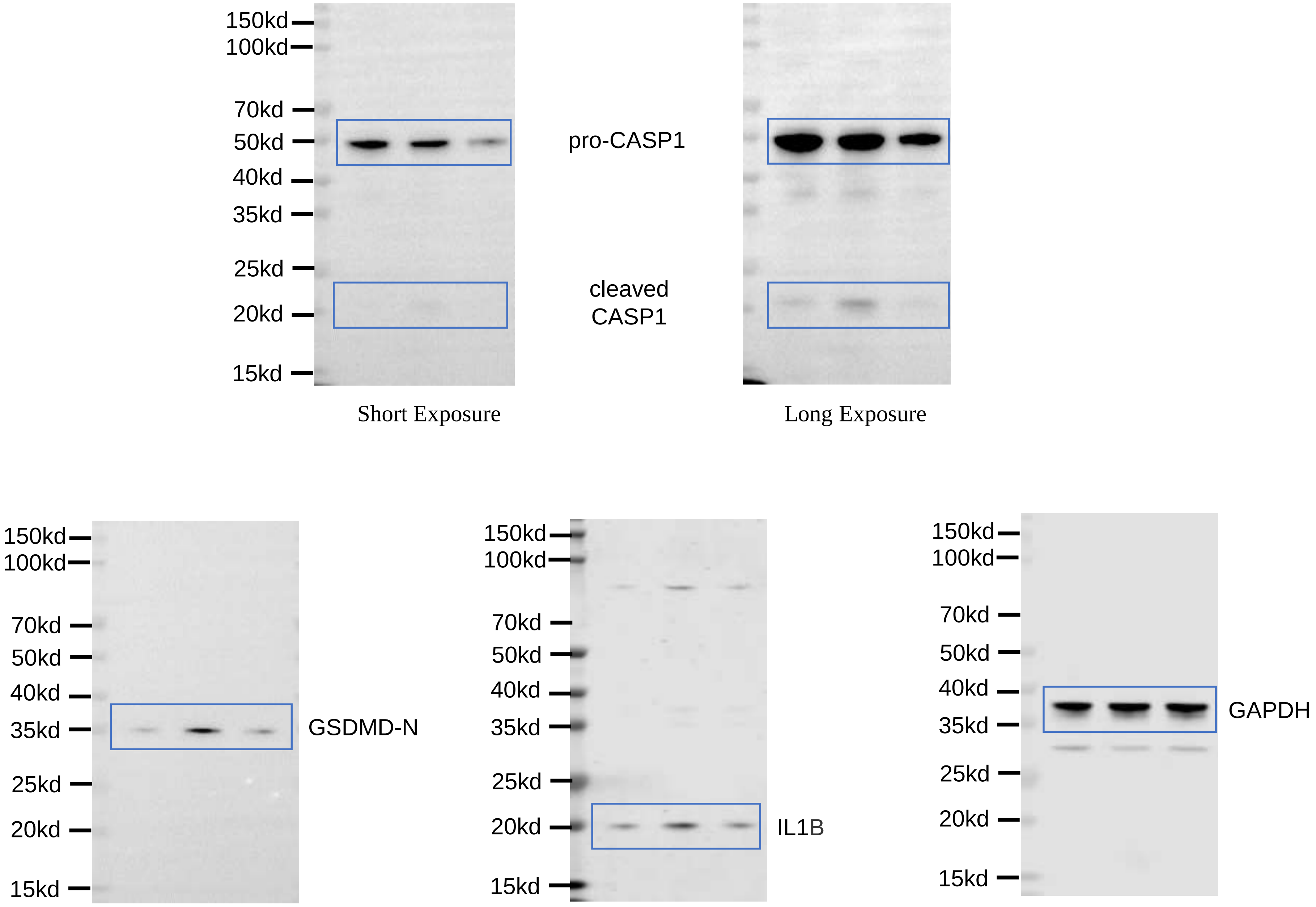

Uncropped versions of blots shown in Figure 2A

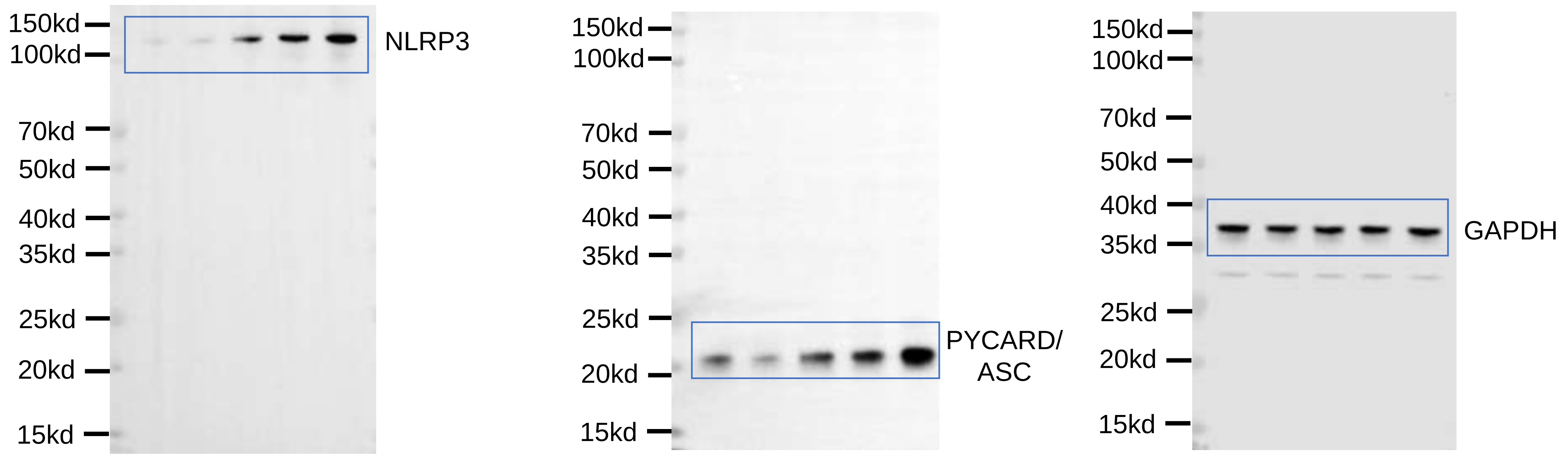

Uncropped versions of blots shown in Figure 3A

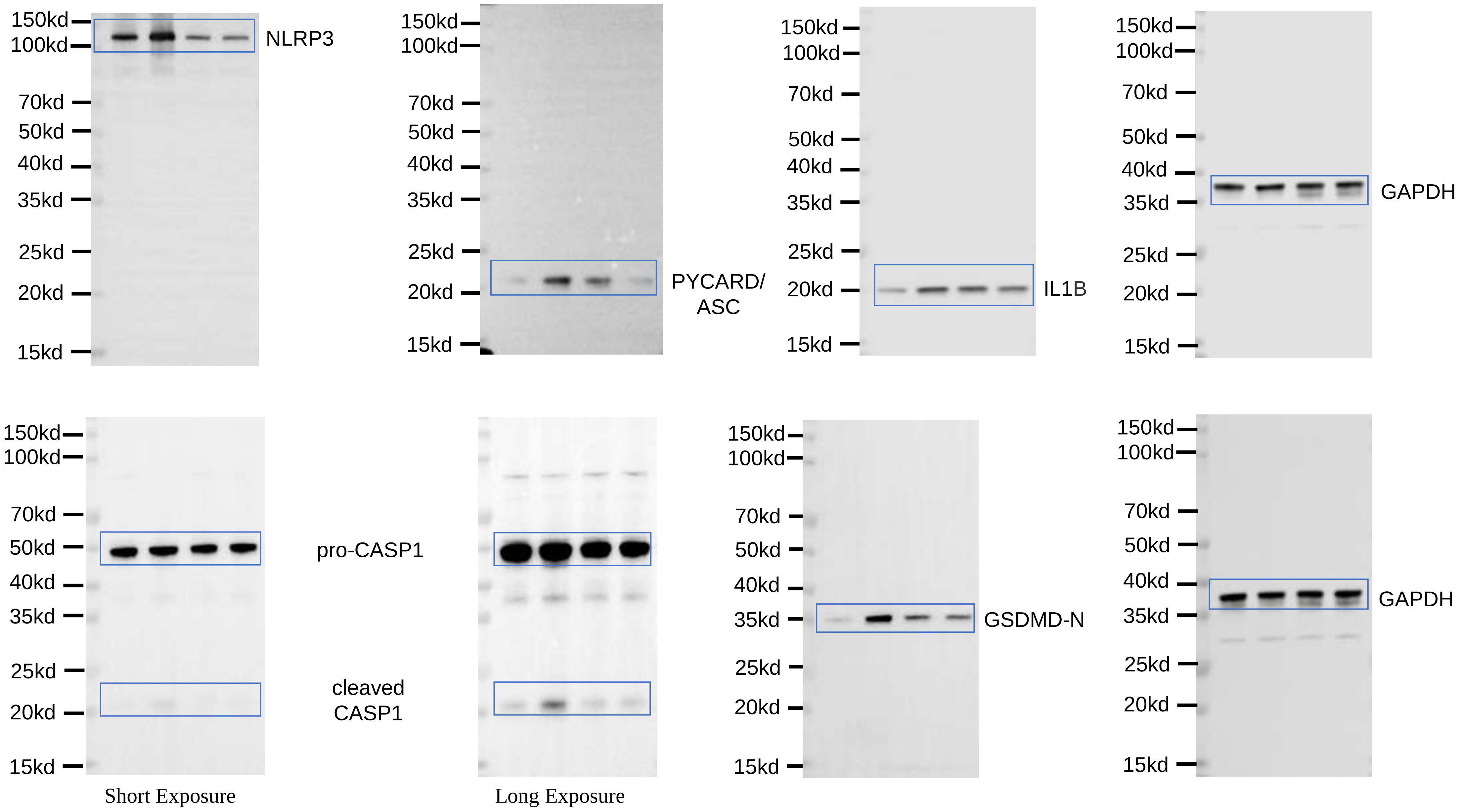

Uncropped versions of blots shown in Figure 3C

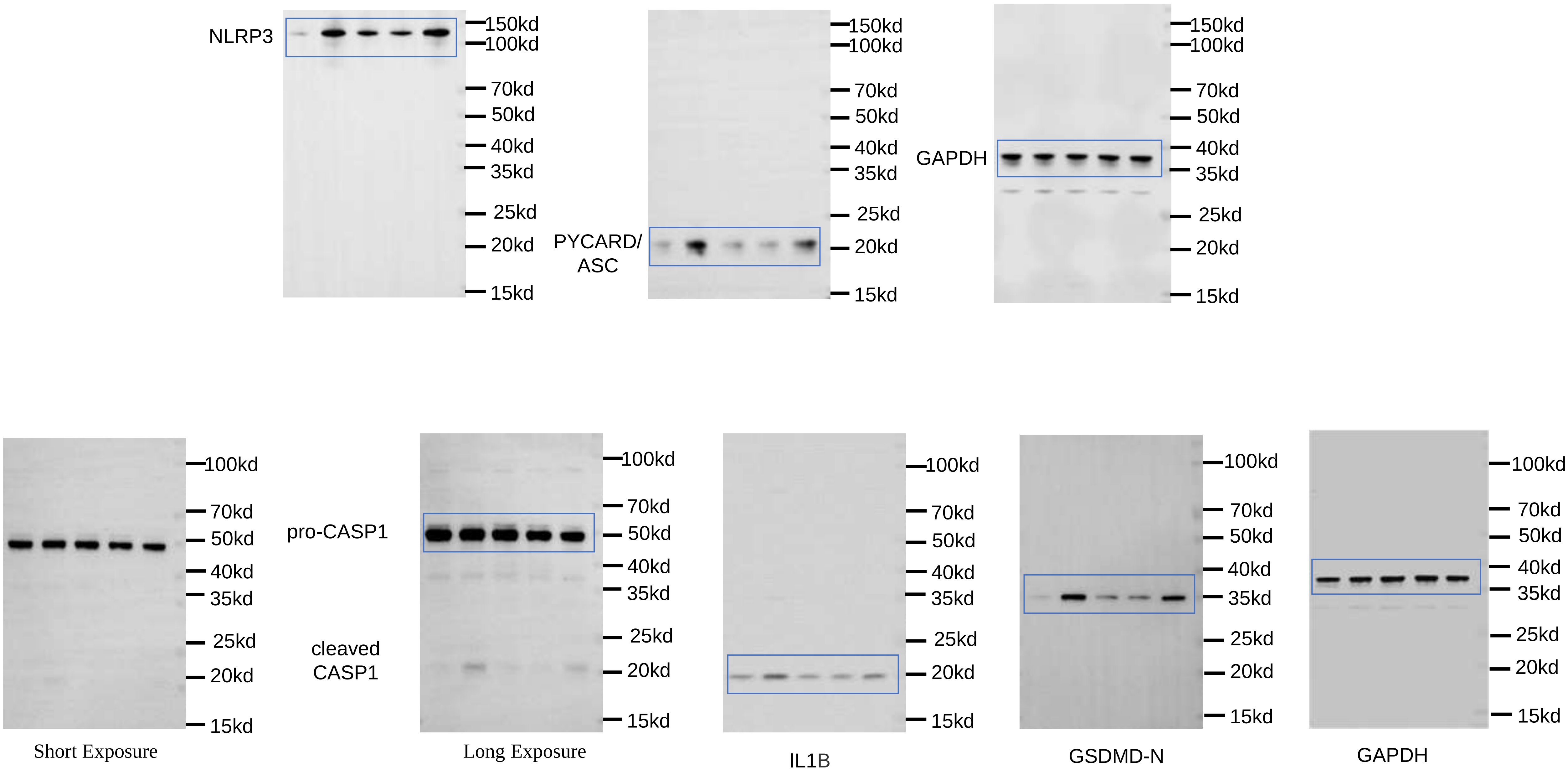

Uncropped versions of blots shown in Figure 4A

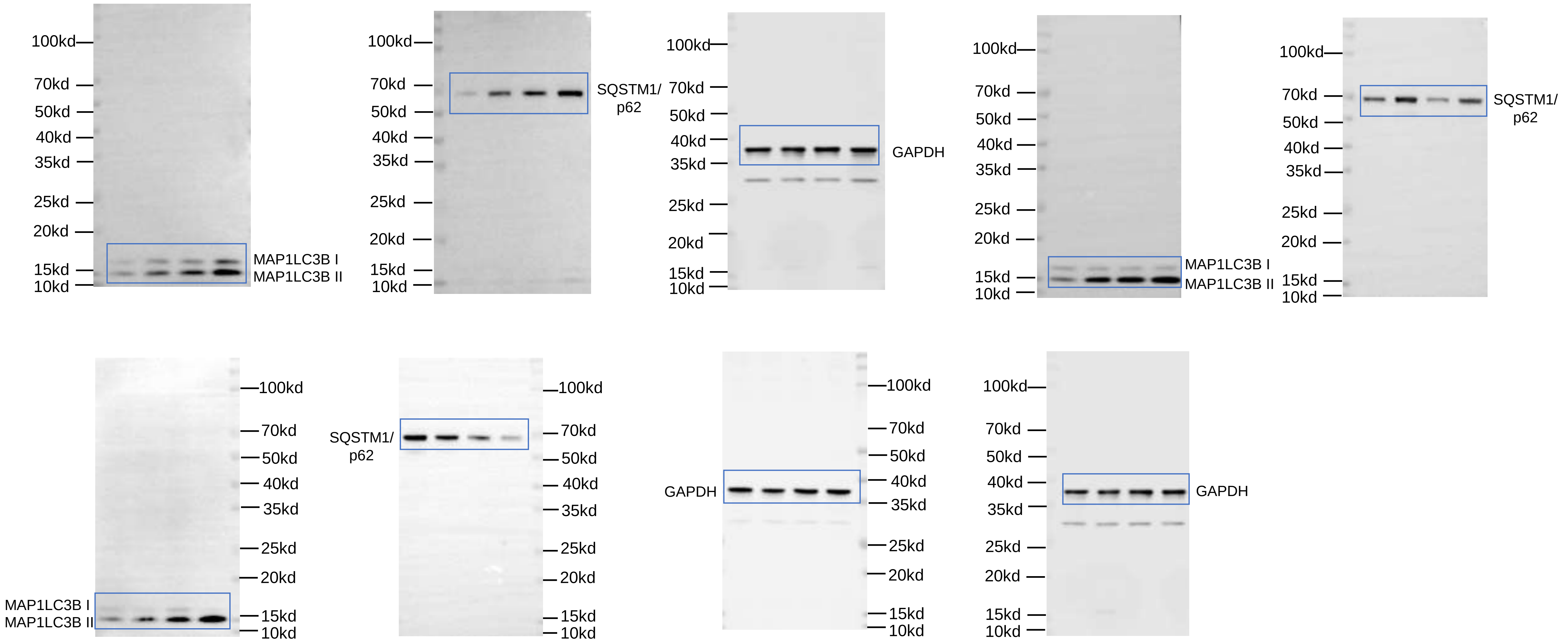

Uncropped versions of blots shown in Figure 5C,E,G

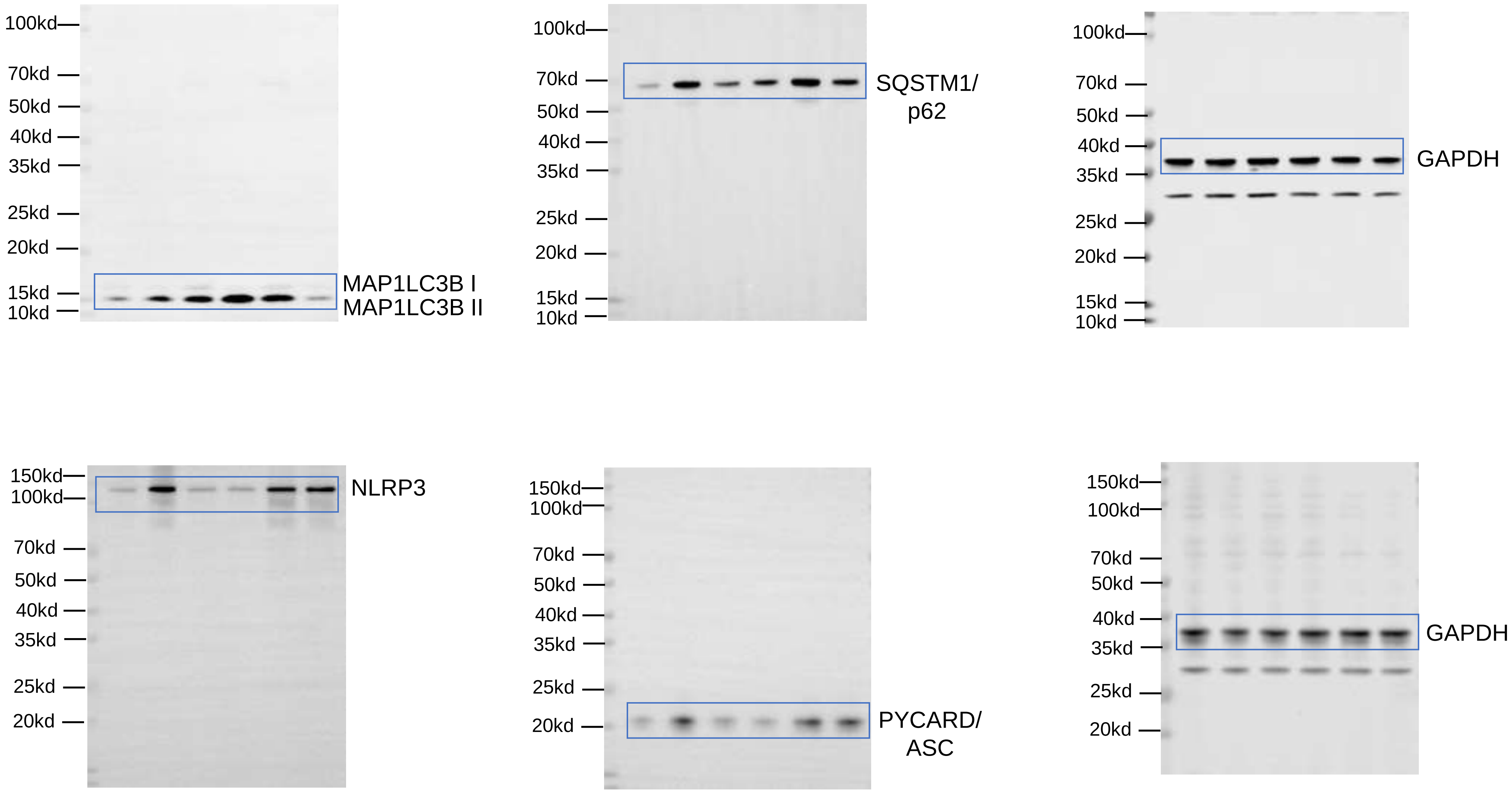

Uncropped versions of blots shown in Figure 6A

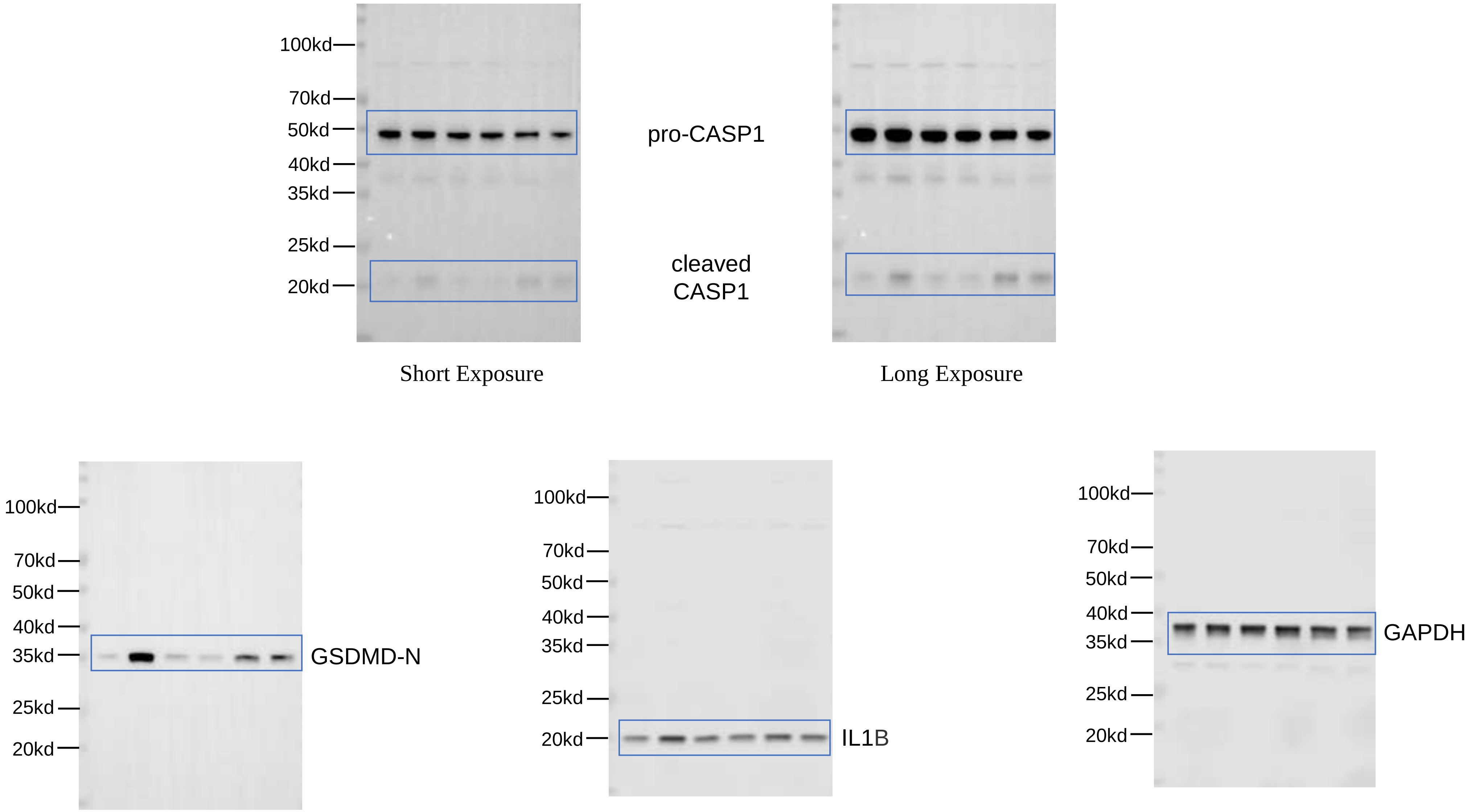

Uncropped versions of blots shown in Figure 6A

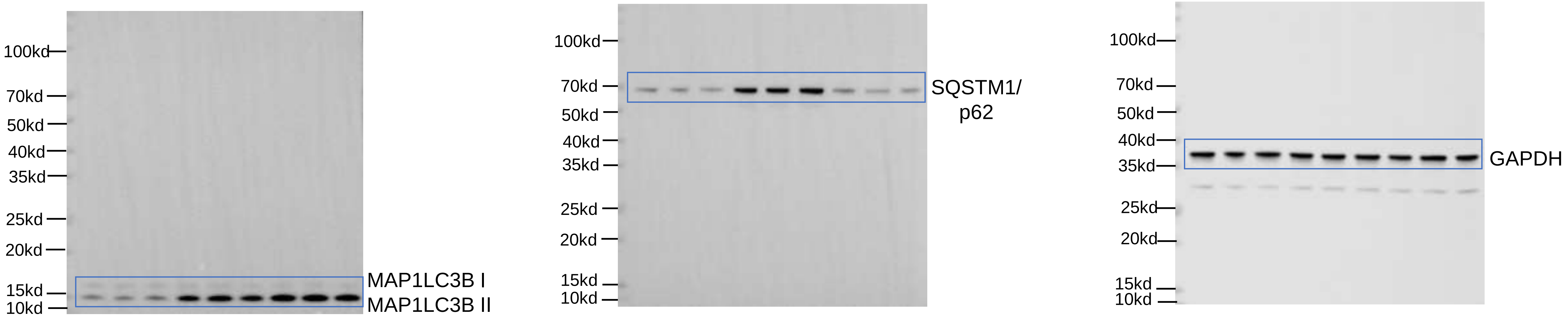

Uncropped versions of blots shown in Figure 7J

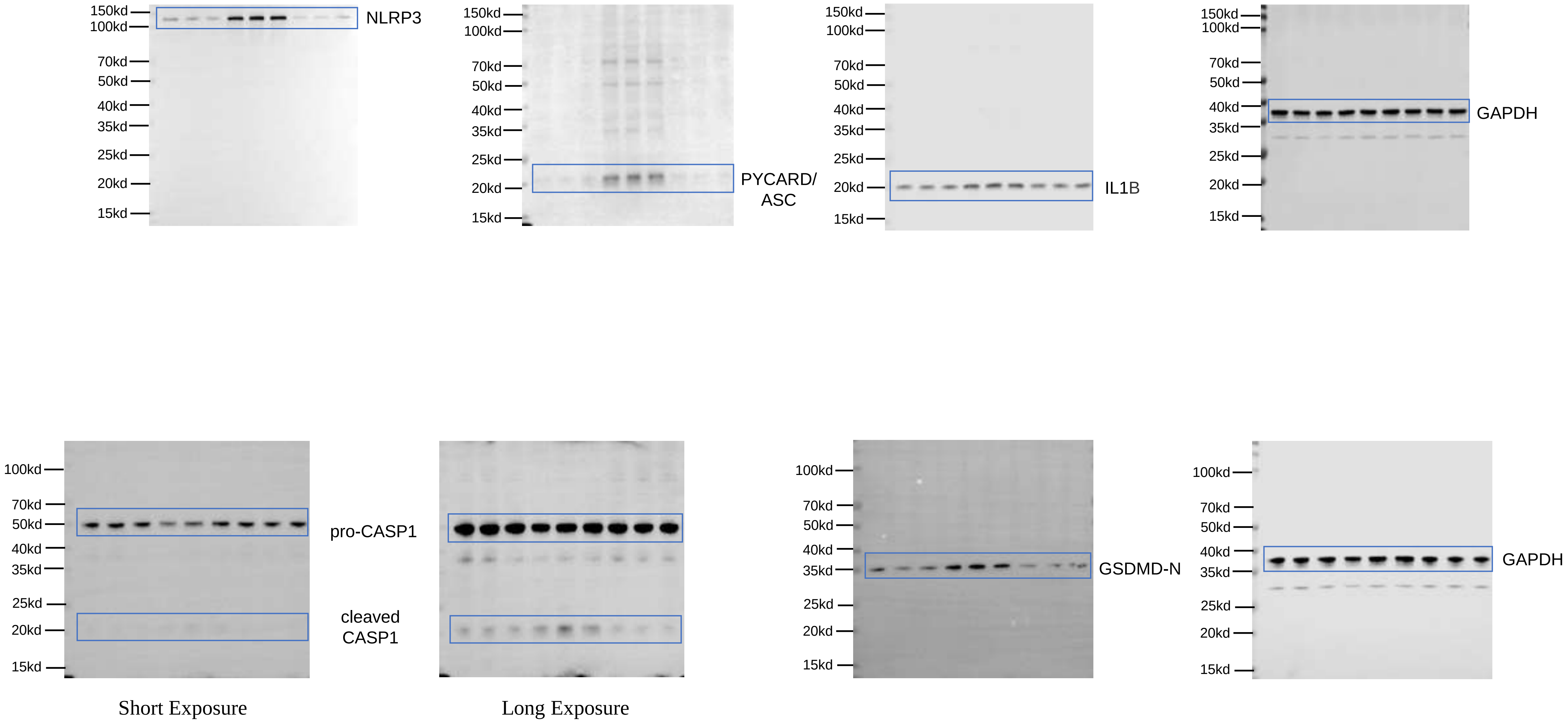

Uncropped versions of blots shown in Figure 7J

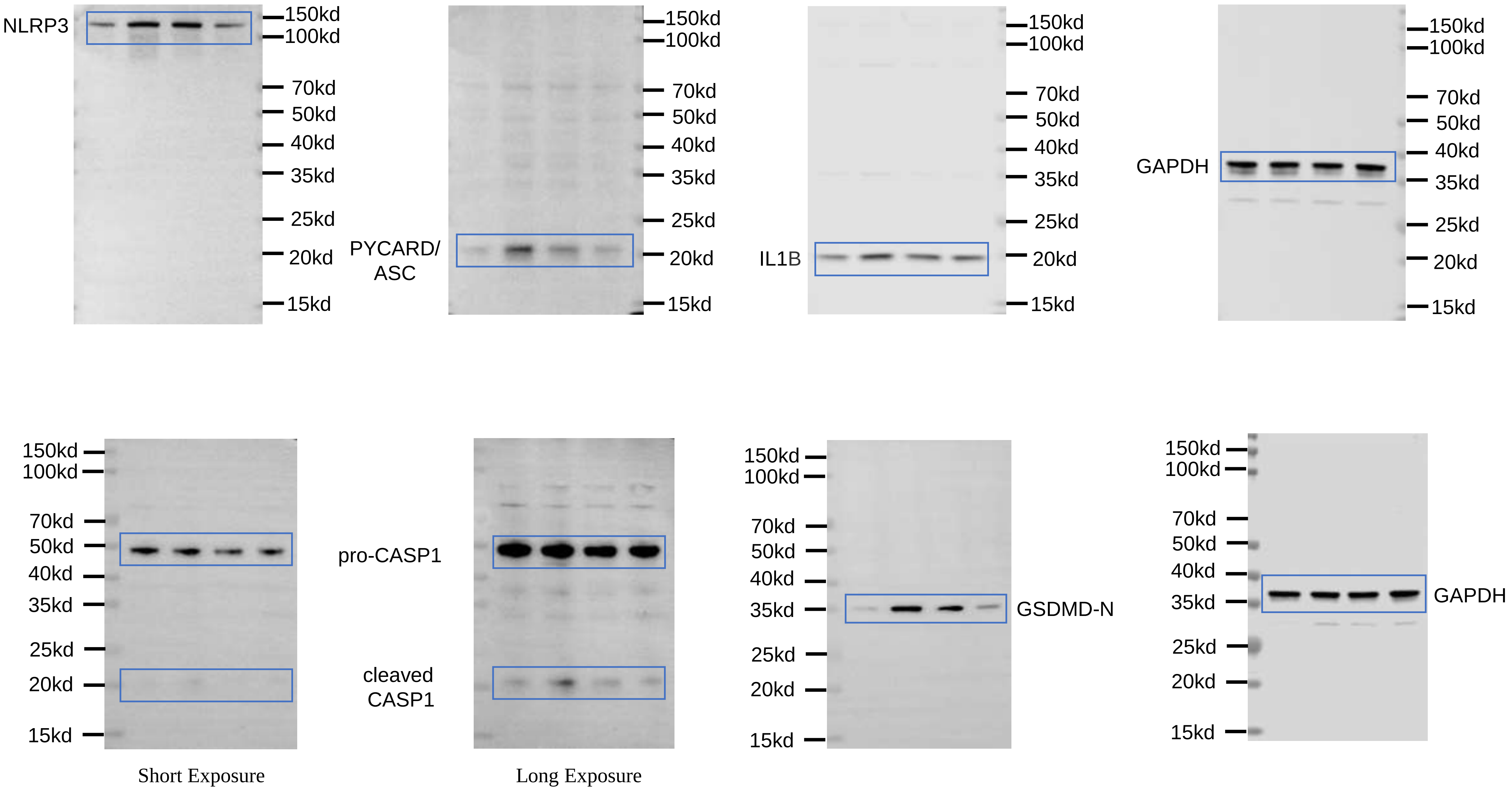

Uncropped versions of blots shown in FigureS1C

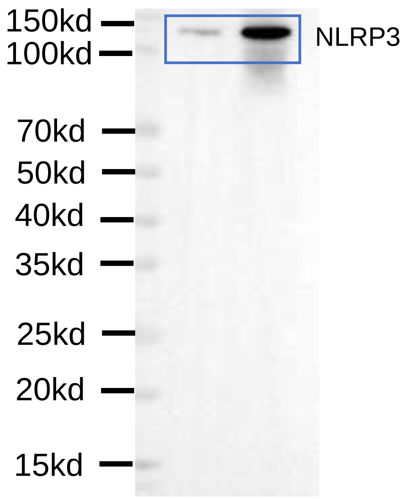

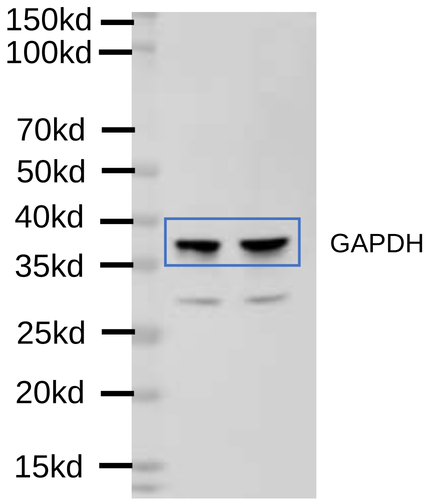

Uncropped versions of blots shown in FigureS2
