# Supplementary material for: Irisin protects against vascular calcification by activating autophagy and inhibiting NLRP3-mediated vascular smooth muscle cell pyroptosis in chronic kidney disease
Source: Cell Death Dis. 2022 Mar 30;13(3):283. doi: 10.1038/s41419-022-04735-7 (PMC8967887; doi:10.1038/s41419-022-04735-7)
Supplement: Supplementary file 5 — Supplementary Figure legends [file 41419_2022_4735_MOESM5_ESM.docx]

**Fig.S1 Irisin protects VSMCs from β-GP-induced pyroptotic cell death and calcification.** VSMCs were treated with different concentrations of Irisin (Irisin 1: 50 ng/ml; Irisin2：100 ng/ml) for 7 days in medium containing β-GP (10 mM). **A** Calcium deposition in VSMCs was assessed by Alizarin red staining (positive staining: red; scale bar=100 μm). **B** Quantitative analysis of calcium deposition in VSMCs normalized to the protein content. **C** The protein levels of pyroptosis-related markers were determined by western blotting. **D** Quantification of the results shown in C. **E** The IL1B content in VSMC culture supernatants was determined by ELISA. **F** The release of LDH was detected using the LDH Assay Kit. **G** The percentage of PI-positive cells was measured using Hoechst 33342 (blue)/PI (red) double staining (top: Representative images; bottom: Quantitative analysis of PI-positive cells). **H** The percentage of TUNEL-positive cells (green)was evaluated by TUNEL staining (top: Representative images; bottom: Quantitative analysis of TUNEL-positive cells). Scale bar = 50 μm for (G); Scale bar = 20 μm for (H). Data are expressed as mean ± SEM. **P* < 0.05, ***P* < 0.01, ****P* < 0.001 vs. control group; *^#^P*<0.05, *^##^P*< 0.01 vs. β-GP group; and N.S. not significant.

**Fig. S2 The expression level of NLRP3 increases in VSMCs after transfecting the *Nlrp3* overexpression plasmid.** VSMCs were transfected with the pcDNA empty vector or *Nlrp3*-expressed plasmid for 48 h. **A** The mRNA level of *Nlrp3* was validated by qRT-PCR. **B** The protein level of NLRP3 was determined by western blotting. Data are expressed as mean ± SEM.**P* < 0.05, ***P*< 0.01 vs. control group. NLRP3, VSMCs transfected with *Nlrp3*-overexpressed plasmid group; pcDNA, VSMCs transfected with pcDNA empty vector group.
